# Digital selective transformation and patterning of highly conductive hydrogel bioelectronics by laser-induced phase separation

**DOI:** 10.1126/sciadv.abo3209

**Published:** 2022-06-08

**Authors:** Daeyeon Won, Jin Kim, Joonhwa Choi, HyeongJun Kim, Seonggeun Han, Inho Ha, Junhyuk Bang, Kyun Kyu Kim, Youngseok Lee, Taek-Soo Kim, Jae-Hak Park, C-Yoon Kim, Seung Hwan Ko

**Affiliations:** 1 Soft Robotics Research Center, Seoul National University, 1 Gwanak-ro, Gwanak-gu, Seoul 08826, Republic of Korea.; 2 Applied Nano and Thermal Science Lab, Department of Mechanical Engineering, Seoul National University, 1 Gwanak-ro, Gwanak-gu, Seoul 08826, Republic of Korea.; 3 Laboratory Animal Medicine, College of Veterinary Medicine, Seoul National University, 1, Gwanak-ro, Gwanak-gu, Seoul 08826, Republic of Korea.; 4 College of Veterinary Medicine, Konkuk University, 120, Neungdong-ro, Gwangjin-gu, Seoul 05029, Republic of Korea.; 5 Department of Mechanical Engineering, Korea Advanced Institute of Science and Technology (KAIST), Daejeon 34141, Republic of Korea.; 6 Institute of Advanced Machines and Design/Institute of Engineering Research, Seoul National University, Seoul 08826, Republic of Korea.

## Abstract

The patterning of poly(3,4-ethylenedioxythiophene):poly(styrene sulfonate) (PEDOT:PSS) hydrogels with excellent electrical property and spatial resolution is a challenge for bioelectronic applications. However, most PEDOT:PSS hydrogels are fabricated by conventional manufacturing processes such as photolithography, inkjet printing, and screen printing with complex fabrication steps or low spatial resolution. Moreover, the additives used for fabricating PEDOT:PSS hydrogels are mostly cytotoxic, thus requiring days of detoxification. Here, we developed a previously unexplored ultrafast and biocompatible digital patterning process for PEDOT:PSS hydrogel via phase separation induced by a laser. We enhanced the electrical properties and aqueous stability of PEDOT:PSS by selective laser scanning, which allowed the transformation of PEDOT:PSS into water-stable hydrogels. PEDOT:PSS hydrogels showed high electrical conductivity of 670 S/cm with 6-μm resolution in water. Furthermore, electrochemical properties were maintained even after 6 months in a physiological environment. We further demonstrated stable neural signal recording and stimulation with hydrogel electrodes fabricated by laser.

## Teaser

Laser-induced phase separation enabled the rapid transformation of PEDOT:PSS into high-performance conductive hydrogels.

## INTRODUCTION

Engineering conductive hydrogels with excellent electrical properties and aqueous stability is important for developing electrode materials that are of broad interest to research fields such as bioelectronics (*1*), e-skin (*2*), and energy devices (*3*). In bioelectronics, achieving tissue-like mechanical properties and retaining their electrical conductivity under strain in physiological environments are crucial for electrode materials to interface with elastic soft tissue in a long-term operation (*4*, *5*).

Hydrogels fabricated by conducting polymers [e.g., polypyrrole, polyaniline, and poly(3,4-ethylenedioxythiophene):polystyrene sulfonate (PEDOT:PSS)] are promising electrode materials (*1*) owing to superior softness, stretchability, and electrochemical stability to metal (Au and Pt) (*6*, *7*) and oxide materials (IrOx and RuO) (*6*) in physiological environments. In particular, conductive hydrogels consisting of PEDOT:PSS have attracted the most attention due to their unique electrical and ionic dual conductivity and excellent biocompatibility. However, PEDOT:PSS is disadvantageous for long-term operation in contact with biological tissues as it suffers from a relatively high Young’s modulus (1 to 2 GPa), low stretchability (~2% strain) (*8*) due to the brittle PEDOT-rich domain, and water instability due to the hydrophilic PSS-rich domain. To transform PEDOT:PSS into water-stable soft hydrogels, phase separation methods that properly redistribute the networks between the conductive and hydrophobic PEDOT-rich domain and the soft and hydrophilic PSS-rich domain have been developed (*4*, *9*–*17*). For phase separation, sulfuric acid or ionic liquids with strong ionic strength were usually introduced to break the ionic bond between positively charged PEDOT (PEDOT^+^) and negatively charged PSS (PSS^−^) and rearrange the networks between them (*4*, *9*–*14*). Subsequent dry annealing was able to physically interconnect the PEDOT-rich domain that made the PEDOT:PSS hydrogel stable in aqueous environments. However, the additives were harmful to living tissues, thus requiring posttreatment of washing with water for several days before usage (*4*, *9*, *11*–*14*). For phase separation, a strong electric field was also applied with several hours of thermal annealing in a state where the ionic bond between PEDOT^+^ and PSS^−^ was weakened by additives (*15*–*17*). Even after tedious and lengthy postprocesses, the hydrogels in previous studies had a low electrical conductivity of less than 200 S/cm when fully swollen in the electrolyte.

Despite the promising properties of PEDOT:PSS hydrogels, patterning them with a high spatial resolution is another major challenge for bioelectronic applications. Various printing methods [e.g., inkjet printing, screen printing, and three-dimensional (3D) printing] (*13*, *14*, *18*, *19*) were developed by mixing additives into PEDOT:PSS aqueous solution that can induce phase separation for transformation into hydrogels. However, most of the studies showed low spatial resolutions of over 100 μm and required a long detoxification process to remove cytotoxic additives. Processes to change the physical properties of PEDOT:PSS by irradiating a light source have also been attempted (*4*, *20*, *21*). The incorporation of a photo–cross-linkable monomer has been tried, but a nonconductive monomer could reduce the conductivity of PEDOT:PSS hydrogel (*4*, *20*). Supplying infrared photons to selectively remove PSS shell for conductivity enhancement has been introduced (*21*); however, it could not induce physical aggregation of PEDOT-rich domain, which cannot assure aqueous stability in physiological environments.

Here, we developed a novel biocompatible and ultrafast digital patterning process to fabricate water-stable PEDOT:PSS hydrogels via the laser-induced phase separation of PEDOT:PSS (LIPSP). We took advantage of the unique characteristics of a continuous-wave laser (*22*), which can provide a strong electric field and photothermal energy in an ultrafast time scale to induce the phase separation of PEDOT:PSS. To intensify the interaction between PEDOT:PSS and the laser, plasmonic gold nanoparticles (AuNPs) (*23*) were added, and the degree of phase separation according to the AuNP concentration was investigated. Through the LIPSP, electrical conductivity and aqueous stability were greatly enhanced owing to the expanded and connected PEDOT-rich domains. The laser-treated PEDOT:PSS was immersed in water so that only the scanned parts remained as water-stable PEDOT:PSS hydrogels, and the rest was washed out. The electrical conductivity of the resulting PEDOT:PSS hydrogels was up to 670 S/cm in the swollen state in the deionized (DI) water with 6-μm spatial resolution. With different volume fractions of AuNP inks to the optimum electrical conductivity, PEDOT:PSS hydrogel also achieved maximum water contents of 39% with a conductivity of 560 S/cm. The excellent aqueous stability of the laser-treated PEDOT:PSS hydrogel allowed the electrochemical properties to be maintained even after immersion in phosphate-buffered saline (PBS) for 6 months. The LIPSP was completely sterile and did not require toxic additives, eliminating the need for a multiday detoxification process. Excellent in vitro and in vivo biocompatibility was confirmed by testing the samples immediately after the laser process without a postcleaning process. To demonstrate the potential of our laser process for bioelectronic fabrication, representative bioelectronic applications were performed. Neural probes with high spatial resolution stably recorded neural signals from a brain slice of mice. Also, a highly conductive electrode array was able to stimulate the sciatic nerve of mice with low voltage.

## RESULTS

### Laser-induced phase separation of conducting polymer

**Fig. 1.**
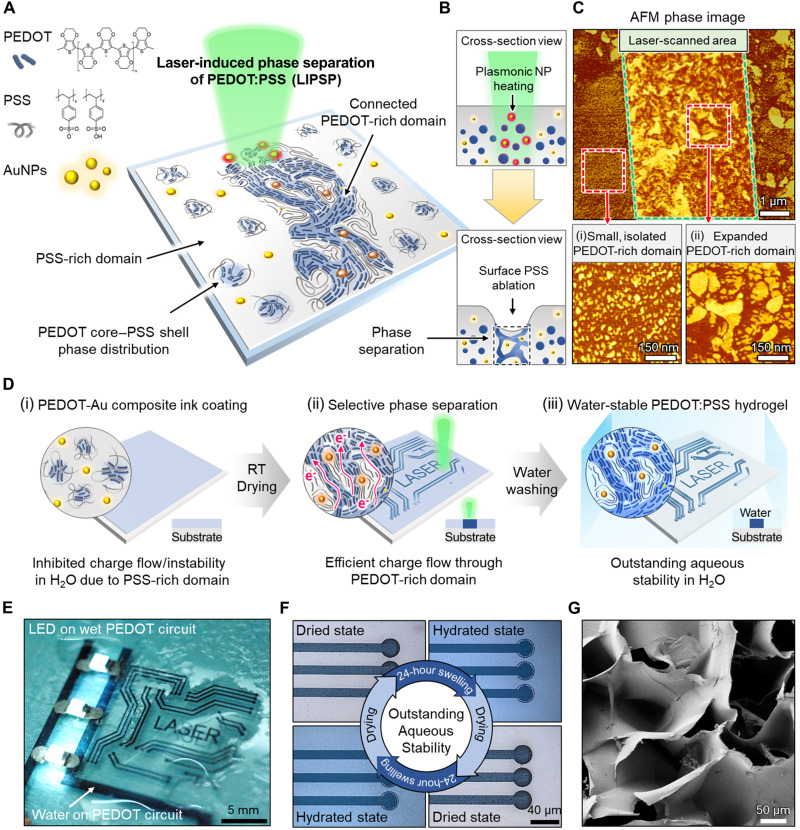
Laser-induced phase separation of conducting polymer. (**A**) Schematic illustration of the LIPSP. (**B**) Photothermal effect induced by interactions between the laser and plasmonic AuNPs (top) and ablation of excessive surface PSS and phase separation (bottom). (**C**) Atomic force microscopy (AFM) phase image of the LIPSP (top, inside green dotted area = laser-scanned area) with magnified phase images of the (i) nontreated region and (ii) laser-scanned region (bright color = PEDOT-rich domain; dark color = PSS-rich domain). (**D**) Fabrication steps for the micropatterning of PEDOT:PSS hydrogels via the LIPSP. (**E**) Micropatterned PEDOT:PSS hydrogel circuit turning on three LEDs in the hydrated state. (**F**) Cyclic swelling and drying for aqueous stability testing. (**G**) SEM image of laser-treated PEDOT:PSS hydrogels.

PEDOT:PSS shows a phase distribution where the conducting PEDOT is in the core and the insulating PSS is in the shell (*24*). The PSS shell surrounding the PEDOT core not only blocks charge flow but also contributes to the fragmentation of PEDOT:PSS in the presence of moisture due to its hydrophilic properties (*25*). A key strategy to transform PEDOT:PSS into water-stable hydrogels is to redesign the phase distribution of PEDOT:PSS. Phase separation is a unique approach to fabricate PEDOT:PSS hydrogels by appropriately controlling the arrangement and crystallization of the PEDOT-rich domain and PSS-rich domain (*4*, *9*–*17*). Specifically, a strong interconnection between the conductive and hydrophobic PEDOT-rich domains can provide excellent electrical conductivity and aqueous stability. We developed a novel ultrafast digital patterning process to fabricate highly conductive PEDOT:PSS hydrogels (670 S/cm in the swollen state) via the LIPSP without an additional postchemical removal process (Fig. 1). Considering the enhanced properties of PEDOT:PSS hydrogels attributed to the phase separation of PEDOT:PSS through dry annealing processes (*13*, *14*) and strong electric field (*15*–*17*) reported in previous studies, we hypothesized that a laser can give both photothermal energy and electric field to PEDOT:PSS simultaneously (*22*). The photothermal energy would locally make the PEDOT and PSS two-phase system unstable, and then the strong electric field was able to separate the PEDOT and PSS. Then, since PEDOT:PSS was rapidly cooled down to room temperature after laser scanning, the phase distribution of PEDOT:PSS was fixed in a separated state (Fig. 1A). AuNP inks using ethanol as a solvent were blended with PEDOT:PSS to enhance laser absorption (*23*), allowing PEDOT and PSS to be separated and recrystallized through rapid temperature elevation and an amplified electric field (Fig. 1B and fig. S1). Selective phase separation by the laser was confirmed with atomic force microscopy (AFM) phase image analysis (Fig. 1C and fig. S2). In comparison with the small PEDOT-rich domain (bright color) covered by the PSS-rich domain (dark color) before laser irradiation (Fig. 1C, i), the PEDOT-rich domain was greatly expanded and connected after the LIPSP (Fig. 1C, ii). The strong network between the PEDOT-rich domains could form a stable mechanical connection in water, and the expanded PEDOT domain could increase the pathway for rapid charge flow (*13*). To fabricate micropatterned PEDOT:PSS hydrogels, PEDOT:PSS and AuNP composite inks were coated onto a substrate through a solution process such as spin coating and drop casting, followed by drying at room temperature (Fig. 1D, i). Phase separation was selectively induced by high-speed laser scanning (Fig. 1D, ii, and movie S1). In this case, only the parts scanned by the laser became highly conductive, and the water stability was greatly increased. After immersing the sample in water and waiting for 5 min, only the laser processed region remained, and the rest were dissolved in water to form fine patterns (Fig. 1D, iii, and movie S2). The micropatterned PEDOT:PSS hydrogels demonstrated excellent electrical conductivity, which can turn on the light-emitting diodes (LEDs) under wet conditions (Fig. 1E). Furthermore, owing to the high stability in aqueous environments, the pattern resolution and electrical properties remained intact even after several swelling and drying cycles (Fig. 1F and figs. S3 and S4). To confirm that the micropatterned material was PEDOT:PSS hydrogel, the sample was lyophilized, and its porous characteristics were examined by scanning electron microscopy (SEM) (Fig. 1G).

### Phase separation analysis of conducting polymer

**Fig. 2.**
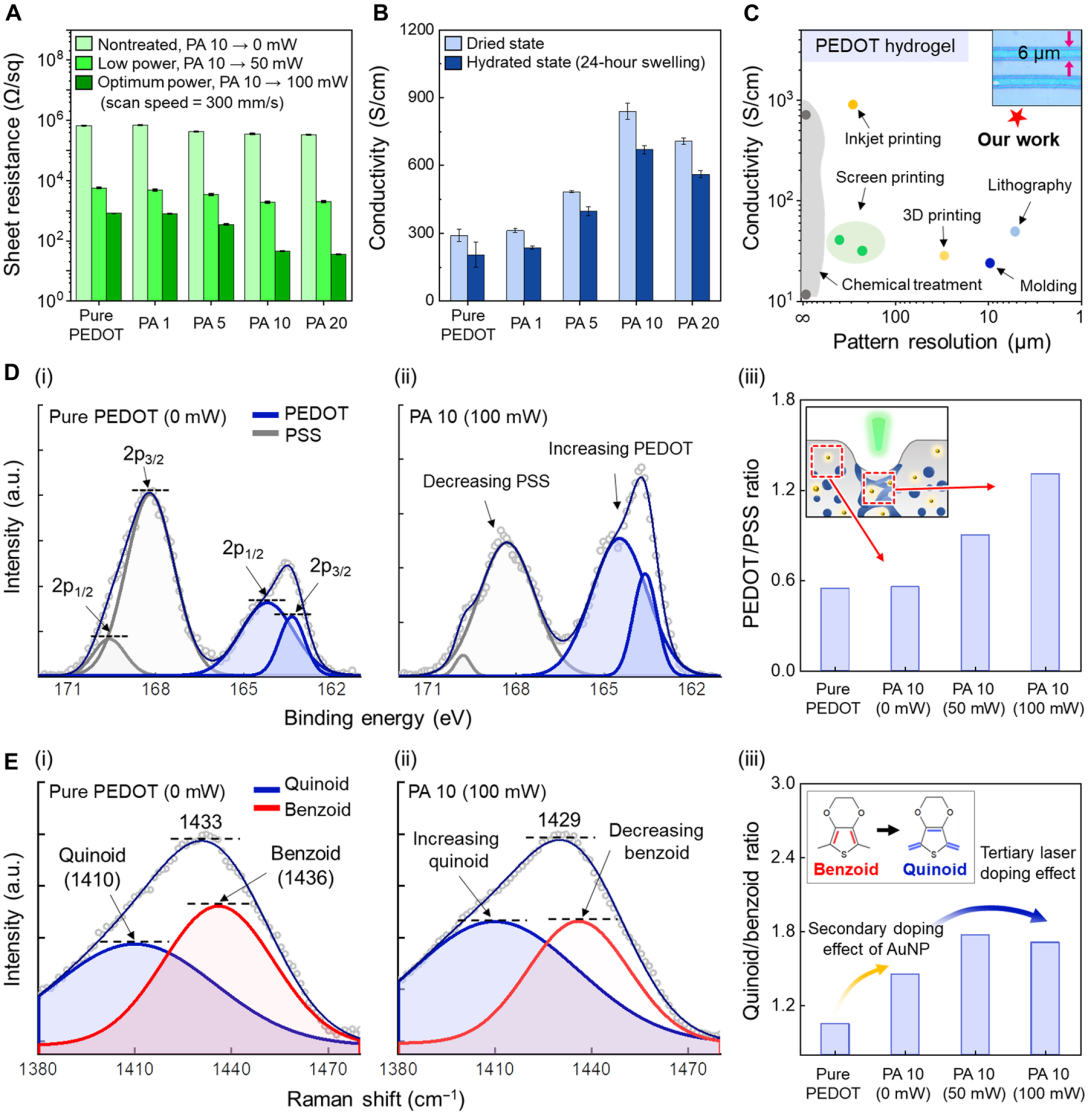
Analysis of laser-treated PEDOT:PSS. (**A**) Sheet resistance of PEDOT:PSS samples according to the AuNP ink volume fraction and laser parameters. (**B**) Electrical conductivity of laser-treated PEDOT:PSS hydrogels in the dried state and hydrated state (24 hours of swelling) according to the AuNP concentration. (**C**) Comparison of the present study with preceding studies in terms of electrical conductivity in the hydrated state and spatial resolution (inset shows an optical microscope image of 6-μm spatial resolution). (**D**) XPS results for pure PEDOT:PSS and laser-treated PEDOT:PSS. The deconvolution of XPS peaks was performed between 171 and 162 eV, showing a gradual increase in the PEDOT to PSS ratio depending on the laser parameters. (**E**) Raman spectroscopy results for pure PEDOT:PSS and laser-treated PEDOT:PSS. The deconvolution of Raman spectral data was performed between 1380 and 1480 cm^−1^, demonstrating a transformation from the benzoid (1436 cm^−1^) to quinoid (1410 cm^−1^) structure through the LIPSP. Values in (A) and (B) represent the mean, and the error bar represent the SD (*n* = 5).

We investigated the effect of the AuNP ink volume fraction in PEDOT:PSS on the phase separation of the resulting laser-treated PEDOT:PSS. AuNP inks [0.1 weight % (wt %) in ethanol, polyvinyl pyrrolidone (PVP)–stabilized, diameter = 40 to 60 nm] were blended with PEDOT:PSS solution (1.1 wt % in water) at 1 to 20 volume % (denoted as PA 1 to PA 20) of the final PEDOT:PSS and AuNP composite (PA) inks and dried on a substrate. A higher AuNP volume fraction enhanced the absorption of laser and increased the thermal and electric field effects, as shown in decreasing optimum laser power as we increased the volume fraction of AuNP inks (fig. S5). To verify the marked increase of electrical conductivity by the interaction of PEDOT:PSS and the laser, bulk thermal annealing was compared (fig. S6). The mere addition of AuNPs also improved electrical conductivity; however, the effect was un-noticeable since the sheet resistance of bulk thermal annealing was far higher than the laser-treated PEDOT:PSS (fig. S7). Under optimal laser conditions, the electrical conductivity and aqueous stability of PEDOT:PSS were increased with increasing laser power; however, under high-power conditions, PEDOT:PSS became carbonized, and its conductivity started to decrease (Fig. 2A and figs. S5C and S8). For PA 10, combined with the effect of AuNPs, fast laser scanning can achieve the highest electrical conductivity from 1 S/cm in dried pure PEDOT:PSS to 670 S/cm in the fully swollen hydrogel (Fig. 2B). The results showed the high electrical conductivity of micropatterned PEDOT:PSS hydrogels with a high pattern resolution of 6 μm, which is similar to that of photolithography (*4*) involving multiple complex processes (Fig. 2C and table S1). The phase separation of PEDOT:PSS in depth direction was also explored to estimate the processibility of LIPSP for thick PEDOT:PSS treatment (Supplementary Text and fig. S9). The laser intensity at the surface of 10-μm thickness of PEDOT:PSS was enough to induce the phase separation of PEDOT:PSS. Then, we demonstrated that LIPSP was able to uniformly induce the phase separation of PEDOT:PSS in both thin (~500 nm) and thick (~10 μm) PEDOT:PSS films through experiments. In addition, the novel manufacturing process could fabricate conductive hydrogel-based bioelectronics at a fast processing speed compared with other processes, as it does not require lengthy postchemical removing steps (table S2). However, when an excessive amount of AuNPs inks was added, the relative amount of PEDOT:PSS contents became insufficient to form a stable conducting network between the PEDOT-rich domains. In detail, in AuNP inks, PVP stabilizer was used for nanoparticle dispersion. As the volume fraction of AuNP inks was overloaded to PEDOT:PSS hydrogels, the electrical conductivity started to decrease since PVP is an electrical insulator, as observed in the case of PA 20 (Fig. 2B). The PVP contents in PEDOT:PSS hydrogels were characterized by performing Fourier transform infrared (FT-IR) spectroscopy (fig. S10). We further investigated the degree of phase separation of PA 10 compared with pure PEDOT:PSS samples by varying the laser parameters. X-ray photoelectron spectroscopy (XPS) analysis revealed two peaks between 167 and 162 eV, which indicated the S(2p) peaks of sulfur atoms in the PEDOT chain (blue line), and the S(2p) peaks of the PSS chain could be found in the range of 171 to 166 eV (gray line) (Fig. 2D) (*25*, *26*). The relative peak intensity ratio of PEDOT to PSS was calculated by integrating the areas under each peak based on XPS fitting data (Fig. 2D, i and ii). There was no notable change in the ratio by simply adding AuNPs; however, it was gradually increased as the laser power was increased (Fig. 2D, iii, and fig. S11). The ratio of pure PEDOT:PSS without laser treatment [pure PEDOT (0 mW)] was 0.56, whereas that of PA 10 treated with a laser power of 100 mW was 1.31, which explains the enhancement of electrical conductivity and aqueous stability owing to expanded and connected PEDOT-rich domains. We also performed Raman spectroscopy to investigate the molecular state of the PEDOT-rich domain (Fig. 2E). The chemical structure of the PEDOT crystallite is composed of a benzoid structure (1436 cm^−1^) with low electrical conductivity and a quinoid structure (1410 cm^−1^) with high electrical conductivity (*26*, *27*). The transition from the benzoid to quinoid structure indicated a conformation change in the PEDOT morphology from a coiled structure to an extended coil or linear structure, which may be referred to as the secondary doping effect (*27*). We confirmed the doping effect of the LIPSP through the red shift (from 1433 to 1429 cm^−1^) of the C_α_ = C_β_ stretching vibration of thiophene rings between 1380 and 1470 cm^−1^ (Fig. 2E, i and ii, and fig. S11). The relative peak intensity ratio of the quinoid to benzoid structure was also calculated by integrating the areas under each curve based on Raman spectroscopy fitting data (Fig. 2E, i and ii). In addition to the secondary doping effect of ethanol in AuNP inks (*28*) [increased from pure PEDOT (0 mW) = 1.05 to PA 10 (0 mW) = 1.45], fast laser scanning had an additional doping effect, which we define as the tertiary laser doping effect [PA 10 (100 mW) = 1.71] (Fig. 2E, iii).

### Characterization of PEDOT:PSS hydrogel under strain in an aqueous environment

**Fig. 3.**
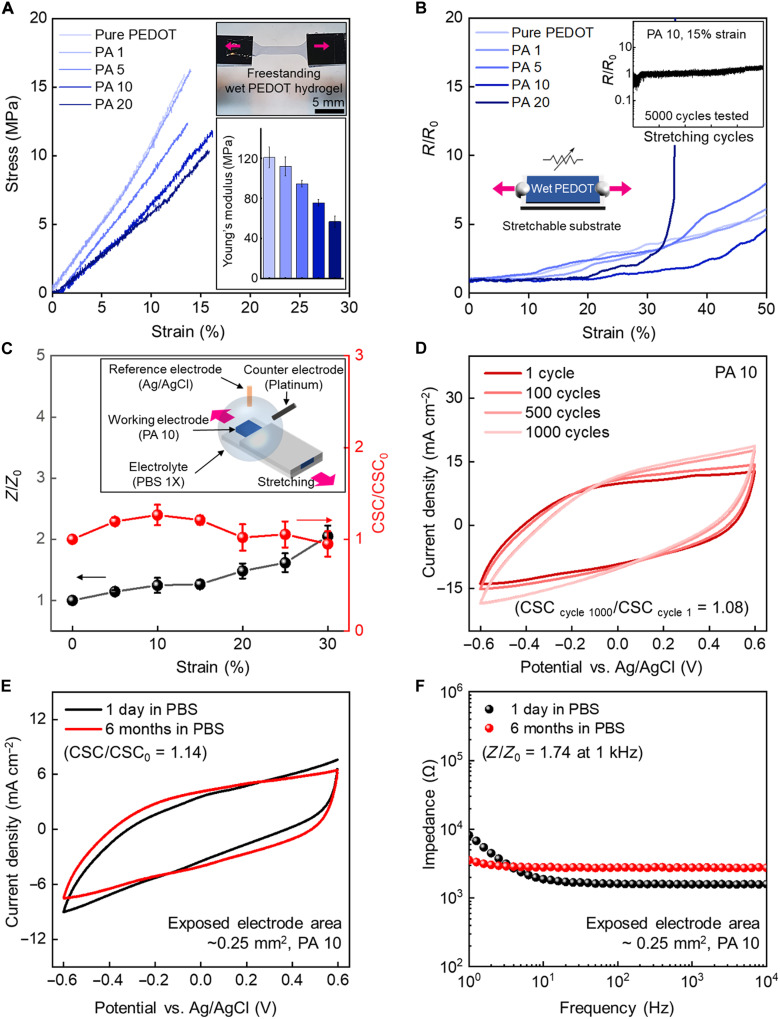
Characterization of PEDOT:PSS hydrogel in an aqueous environment. (**A**) Tensile testing of laser-treated PEDOT:PSS hydrogels in water (inset image), stress-strain curves of PEDOT:PSS hydrogels according to the AuNP volume fraction (main graph), and Young’s modulus of PEDOT:PSS hydrogels (inset graph). (**B**) Electrical resistance of PEDOT:PSS hydrogels under strain according to the AuNP volume fraction (inset shows the cyclic stretching test under 15% strain with 5000 stretching cycles for the PA 10 sample). (**C**) Electrochemical properties of the PA 10 sample under strain [working electrode = PA 10, counter electrode = Pt plate, and reference electrode = Ag/AgCl (3 M KCl)]. (**D**) CV cyclic test of the PA 10 sample. (**E** and **F**) Long-term stability characterization of PEDOT:PSS hydrogels in PBS for 6 months (CV and EIS). Values in (A) and (C) represent the mean, and the error bar represents the SD (*n* = 3).

Owing to enhanced aqueous stability with the LIPSP, we performed tensile tests of free-standing PEDOT:PSS hydrogels under DI water (Fig. 3A). Dried PEDOT:PSS on a substrate with low adhesion (*13*) [e.g., polydimethylsiloxane (PDMS) and glass] was irradiated in the form of dog bone shape through the LIPSP, leaving only the scanned part as a free-standing hydrogel in the water (fig. S12). We measured Young’s modulus of thin PEDOT:PSS hydrogel films using this technique as opposed to nanoindentation, which measures local mechanical properties with high uncertainty (*29*). The stress-strain curve revealed the synergetic effects of AuNP inks and the laser for softening PEDOT:PSS hydrogels by appropriately redistributing the networks between the PEDOT-rich and soft PSS-rich domains. In addition, increasing the AuNP concentration demonstrated the auxiliary effect of adding the PVP stabilizer as interpenetrating polymer networks, which made the composite softer and better to be stretched. PA 20 could achieve an elongation of 20% (fig. S13) and Young’s modulus of 57 MPa, which indicated sufficient softness compared with metal electrodes (*30*) (E_Au_ = 67 GPa, elongation = 2%, tensile tested on water surface), thus minimizing mechanical mismatch when in contact with biological tissues (*31*) (*E*_tissues_ under 1 MPa) for a long period. The swelling ratio and water contents were also shown to demonstrate the swelling behavior of PEDOT:PSS hydrogels under physiological environments (Supplementary Text and figs. S14 and S15). PEDOT:PSS hydrogels showed obvious anisotropic swelling behavior; thus, the swelling ratio and water contents were calculated by measuring the thickness change of PEDOT:PSS micropattern. As the volume fraction of AuNP inks increased, the swelling was enlarged because of the increase in phase separation and hydrophilic PVP contents. Ensuring stable electrical properties under strain is essential for hydrogel-based bioelectronic devices that interface with biological tissues undergoing mechanical deformation. We investigated the resistance change of fully hydrated laser-treated PEDOT:PSS hydrogels under strain by varying the volume fraction of AuNP inks (Fig. 3B). Electrical stability under mechanical deformation was improved with increasing AuNP concentrations; however, in the case of PVP overload (PA 20), the electrical interconnection between the PEDOT-rich domains became unstable due to the unnecessary and excessive PVP contents, which are electrical insulators. The tensile test was repeated on the most electrically stable sample (PA 10), and the resistance remained stable even after 5000 cycles at 15% strain (Fig. 3B, inset graph). We further characterized the electrochemical properties of the PEDOT:PSS hydrogels through electrochemical impedance spectroscopy (EIS) and cyclic voltammetry (CV) (Fig. 3C and fig. S16). The impedance (*Z*) and charge storage capacity (CSC) were measured in PBS, which demonstrated stability up to 30% strain. Excellent electrical and electrochemical properties under mechanical deformation would ensure the stable operation of bioelectronic devices in dynamic environments.

### Long-term stability of PEDOT:PSS hydrogel in an aqueous environment

Maintaining the mechanical and electrical properties of PEDOT:PSS hydrogels in an aqueous environment is crucial for the long-term operation of bioelectronic devices (*4*, *5*). We investigated the stability of PEDOT:PSS hydrogels in physiological environments (PBS). The CSC, which is the performance indicator of bioelectronics where the electrodes can store the electric charges, was maintained with no substantial change even after 1000 repeated CV cycles (average CSC of 32.13 mC cm^−2^, CSC cycle 1000/CSC cycle 1 = 1.08) (Fig. 3D). The charge injection capacity, which is a performance indicator of electrode for electrical neural stimulation, was also characterized. It showed a high charge injection capacity of 16.17 mC cm^−2^ and stability after 10,000 cycles of pulses (fig. S17). Furthermore, EIS and CV were performed after incubating the PA 10 sample in vials for 6 months (Fig. 3, E and F). The results showed that the electrochemical properties were well maintained (CSC/CSC_0_ = 1.14, *Z*/*Z*_0_ = 1.74), making it highly suitable for long-term bioelectronic operation.

### Biocompatibility of PEDOT:PSS hydrogel

**Fig. 4.**
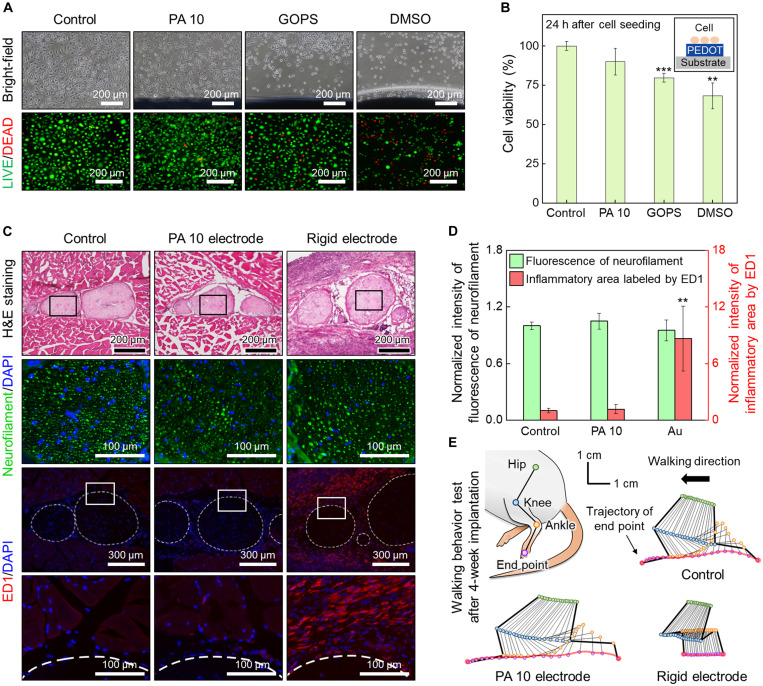
Biocompatibility and behavior analysis of PEDOT:PSS hydrogel. (**A**) Microscopic images of L929 cells at 24 hours seeded on PEDOT:PSS hydrogels. Bright-field microscopy images (first row) and fluorescent microscopy images of live/dead staining (second row). (**B**) MTT assay quantification of cell viability at 24 hours. **P < 0.01 and ***P < 0.001 (*n* = 3, unpaired, two-tailed Student’s *t* test, compared with control). (**C**) Cross-sectional slices of the sciatic nerve implanted with the control, PA 10, and rigid electrodes. H&E staining (first row), fluorescent images immunochemically labeled by the biomarker neurofilament (NF) and 4′,6-diamidino-2-phenylindole (DAPI) (second row), fluorescent images immunochemically labeled by the inflammatory biomarker ED1 and DAPI (third row), and magnified images of fluorescent images immunochemically labeled by ED1 and DAPI (fourth row). (**D**) Histogram showing the normalized fluorescence intensity of NF and ED1 for the control, PA 10, and rigid groups. **P < 0.01 (*n* = 4, unpaired, two-tailed Student’s *t* test, compared with control). (**E**) Hindlimb stick diagram and comparison of kinematic features during walking after implantation with the control, PA 10, and rigid electrodes.

In addition to the excellent electrical properties and aqueous stability of laser-treated PEDOT:PSS hydrogels, biocompatibility is essential for bioelectronic applications. For newly developed bioelectronic materials, various evaluation tests are required to ensure safety (*32*). FT-IR spectroscopy was performed to characterize the ethanol residue from AuNP inks. It showed the complete disappearance of ethanol after just drying the PEDOT:PSS and AuNP composite inks at room temperature (fig. S18). We further investigated the “acute” cell toxicity of PEDOT:PSS hydrogels through in vitro experiments. PA 10 samples were compared with PEDOT:PSS hydrogel samples prepared by blending 1 wt % of (3-glycidyloxypropyl)trimethoxysilane (GOPS) and 13 volume % of dimethyl sulfoxide (DMSO), which are additives used for PEDOT:PSS hydrogel fabrication (*7*, *13*, *14*). Before use in bioelectronic applications, several days of cleaning processes were required to remove these additives due to their cytotoxicity (*4*, *7*, *13*, *14*). In contrast, PA 10 has an advantage in that it does not require additional cleaning steps owing to the aseptic nature of the laser process. To confirm this, all samples were detoxified for 1 hour after each annealing procedure and immediately used for toxicity evaluation. When observing the borderline area in contact with the sample, the cell density and shapes of PA 10 group were similar to the control group. On the other hand, in the GOPS (1 wt %) and DMSO (13 volume %) group, the cells changed to a round shape or fell from the bottom (Fig. 4A, first row). A live/dead cell assay was also performed, and the density of live cells with green fluorescence was similar between the control and PA 10 groups; however, a higher number of dead cells with red fluorescence and surviving cells with a round shape were observed in the GOPS and DMSO groups (Fig. 4A, second row). The 3-(4,5-dimethylthiazol-2-yl)-2,5-diphenyltetrazolium bromide (MTT) assay was performed to measure cell viability, which showed that viability was notably decreased by 20.2% for the GOPS group and 31.6% for the DMSO group compared with the control group (Fig. 4B).

Narrowing the mechanical mismatch between bioelectronic materials and biological tissues is crucial for chronic neuromodulation (*4*, *5*, *32*). It has been reported that rigid implants induce fibrotic and inflammatory reactions in surrounding tissues (*4*, *32*) and can even cause nerve damage in growing animals (*5*). We evaluated the in vivo responses of electrode implantation by comparing soft PEDOT:PSS hydrogel-based electrodes fabricated on soft styrene-butadiene-styrene (SBS) substrate with rigid conventional cuff electrodes fabricated by Au thin film on stiff polyethylene terephthalate (PET) substrate. The electrodes were implanted in mice, and changes in the tissues were analyzed after 4 weeks. No significant difference was observed in neurofilament staining, which indicated minimal nerve damage for all samples (Fig. 4C, first and second rows). However, when the level of inflammation was analyzed using the ED1 biomarker, the inflammatory signal around the nerve bundle was increased for the rigid electrode (Fig. 4C, third and fourth rows). As shown in the graph of the normalized inflammatory signal, the signal of the PA 10 electrode was comparable to that of the control and was significantly lower than that of the rigid electrode (Fig. 4D).

Last, hindlimb kinematic analysis was performed to analyze gait behavior after 4 weeks of implantation. The control and PA 10 electrodes showed normal gait and movement of the joints based on the trajectories of the end points. However, in animals implanted with the rigid electrode, the trajectory was notably changed, and the stride length was reduced (Fig. 4E and movie S3). These results are consistent with typical findings for sciatic nerve injuries or inflammatory responses (*33*, *34*). Together, PEDOT:PSS hydrogels fabricated by the LIPSP, which showed low cytotoxicity and mechanical mismatch, might be an excellent bio-interfacing electronic material.

### Bioelectronic applications

**Fig. 5.**
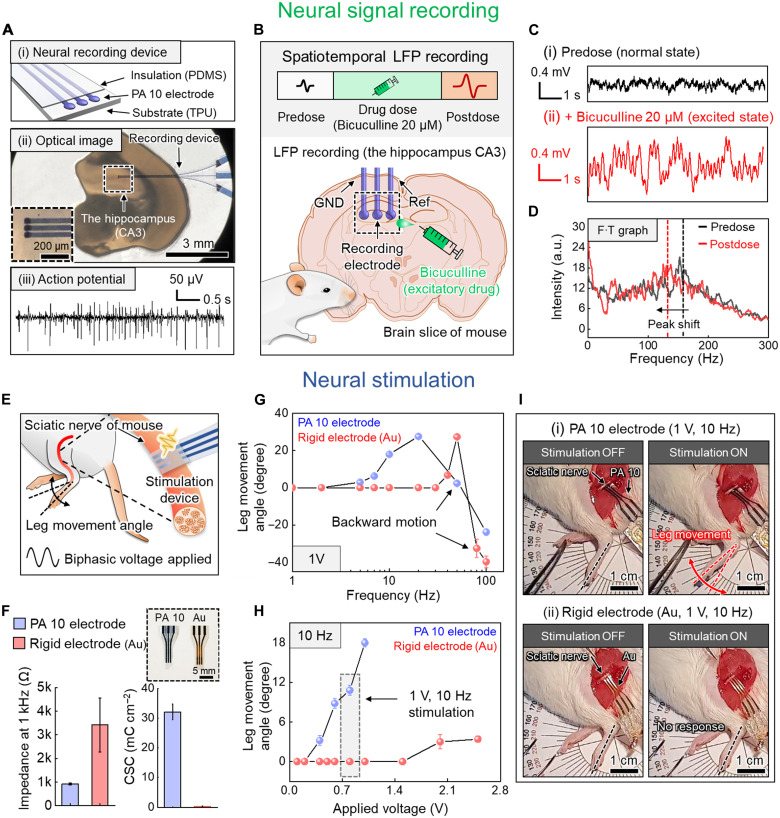
Bioelectronic applications. (**A**) (i) Schematic illustration of the neural recording device based on the PA 10 electrodes and interconnections. (ii) Optical image of the experimental setup for the neural recording of the hippocampus (CA3) of a mouse brain slice (inset image shows the magnified view of three PA 10 electrodes and the hippocampus of the mouse brain). (iii) Extracellular action potential from CA3 of mouse brain slice. (**B**) In situ LFP recording depending on the mouse state. (**C**) LFP signal data without and with bicuculline (predose and postdose). (**D**) Fourier-transformed (F∙T) LFP signal data. (**E**) Schematic illustration of the experiment for neural stimulation of the sciatic nerve of mice. (**F**) Electrochemical properties of stimulation devices (inset shows the real image of stimulation devices). (**G**) Leg movement angle measurement with increasing stimulation frequency and 1 V of amplitude. (**H**) Leg movement angle measurement with increasing stimulation amplitude and 10 Hz of frequency. (**I**) Optical images of leg movement under neural stimulation for devices made using the PA 10 and rigid Au electrodes. (i) Periodic leg movement was observed with the PA 10 electrode at 1 V and 10 Hz; (ii) no response was observed with the rigid Au electrode at 1 V and 10 Hz. Values in (F) to (H) represent the mean, and the error bar represents the SD (*n* = 3).

To verify the potential of the LIPSP as a means of bioelectronic device production, neural signal recording and neural stimulation were performed. The neural signal recording device was fabricated with three PA 10 electrodes (ground, reference, and recording electrodes with 33-μm line width and 10-μm pitch) sandwiched by the thermoplastic polyurethane (TPU) substrate and PDMS insulation layer (Fig. 5A, i, and fig. S19). The hippocampus (CA3) of the brain slice, which can exhibit distinct neural signals (*35*) of the mouse, was gently placed on the recording device (Fig. 5A, ii). We first recorded the extracellular action potential of mouse brain slice, which is the electrical potential that requires a high resolution of microelectrode array for a high signal-to-noise ratio (*36*, *37*). It showed stable recordings of continuous spikes of action potential showing enough potential for future bioelectronic fabrications (Fig. 5A, iii). Furthermore, we recorded the in situ local field potential (LFP), which is the electrical field potential of the extracellular space in the brain tissue (*38*). Excellent electrical properties and fine spatial resolution could allow the stable spatiotemporal recording of LFP signals depending on the state of the mouse brain. An excitatory drug (*39*, *40*) (bicuculline, 20 μM) was treated to the brain slice to induce a change in the mouse state (Fig. 5B). The amplitude of the LFP signal in the normal state (predose) was about 0.16 mV; however, the amplitude after dosing with bicuculline (postdose) was significantly amplified (over 0.4 mV) owing to the excitation of the mouse brain (Fig. 5C). The Fourier-transformed neural signal also showed a marked change in the graph profile after dosing with bicuculline, with a strong signal under a frequency of 12.5 Hz, followed by a steep decline in the range of 17.5 to 33.8 Hz. The peak in the high-frequency range also shifted to a lower frequency from 153 to 130 Hz (Fig. 5D).

For the stimulation of the sciatic nerve of mice, we designed cuff-type stimulation devices using laser-treated soft PEDOT:PSS hydrogels (PA 10 electrode) and rigid type electrodes (Fig. 5E and figs. S19 and S20). Both devices consisted of one ground electrode and two stimulating electrodes passivated by an insulating polymer (0.25-mm^2^ exposed area for both devices). In comparison with the rigid type Au electrode, the PA 10 electrode demonstrated superior electrochemical properties, which is appropriate for low-voltage neural stimulation and can prevent undesired damage to nerve tissues (Fig. 5F) (*4*). The softness of the hydrogel device also had the advantage of conformal contact to the nerve tissue, which was able to deliver efficient charge transfer. The leg movement angle of mice was observed by applying biphasic sinusoidal voltage pulses on the sciatic nerve with varying frequencies and amplitudes. Owing to the low impedance of PEDOT:PSS hydrogels, the movement angle started to increase constantly from a very low frequency (>5 Hz); however, it started to increase at a high frequency (>40 Hz) for rigid Au electrodes (Fig. 5G). The leg started to move backward for both devices when a high stimulation frequency was applied. The threshold voltage at 10 Hz for leg movement was around 0.4 V for the PA 10 electrode; however, it was around 2.0 V for the Au electrode, which can potentially damage nerve tissues (Fig. 5H). Periodic leg movement was observed for the stimulation device made using the PA 10 electrode with stimulation of the sciatic nerve at 1 V and 10 Hz; however, there was no response for the device made using the rigid Au electrode under the same stimulation conditions (Fig. 5I and movie S4).

## DISCUSSION

We present a strategy for the micropatterning of high-performance PEDOT:PSS hydrogels through a laser-induced phase separation process. The developed hydrogels showed high electrical properties with a fine pattern resolution and maintained their electrical and electrochemical properties for an extended period in a physiological environment. The material properties of PEDOT:PSS hydrogels can be tuned by controlling the AuNP concentration and laser parameters. This process is promising for various engineering fields that require electrode materials with excellent aqueous stability in electrolytes and high electrical properties, such as bioelectronics, energy devices, and e-skin devices.

## MATERIALS AND METHODS

### Material preparation

PEDOT:PSS aqueous solution (1.0 to 1.3 wt % solid content, Clevios PH 1000; Heraeus Electronic Materials) was blended with AuNP solution (0.1 wt % PVP-stabilized AuNPs dispersed in ethanol, diameter: 40 to 60 nm; Ditto Technology) at 1 to 20 volume % in the final ink. After stirring for 1 hour at room temperature, the final ink was coated on substrates such as SBS, TPU, and PDMS through solution processes such as spin coating or bar coating and dried at room temperature.

### Laser-induced phase separation of PEDOT:PSS

The laser scanning system consisted of a continuous-wave 532-nm laser (Sprout-G-5W; Lighthouse Photonics), *f*-theta telecentric lens (*f* = 103 mm), and Galvano-mirror (hurrySCAN II; Scanlab), which was used for laser processing to induce selective phase separation. The optimized conditions in this study were 100 mW of laser power and a scanning speed of 300 mm/s for PA 10 (10 volume % AuNPs in the final ink). After laser treatment, the samples were immersed in water for 5 min to wash out nontreated regions.

### Microscopy

Phase and topography images were taken using an atomic force microscope (NX 10; Park Systems). SEM images were obtained using a field-emission scanning electron microscope (SUPRA 55VP; Carl Zeiss) after lyophilization using a freeze dryer (Bondiro; IlShinBioBase). Optical microscopy images were taken using an optical microscope (BX53M; Olympus).

### Material characterization

XPS analysis was conducted using Electron Spectroscopy for Chemical Analysis II (AXIS SUPRA; Kratos). The chemical structure of PEDOT:PSS was analyzed by Raman spectroscopy (inVia Raman microscope; Renishaw), and the absorbance was measured using an ultraviolet-visible (UV-Vis) spectrophotometer (V-770; JASCO).

### Mechanical characterization

Young’s modulus and the elongation until failure were measured by a laboratory-made pseudo–free-standing tensile testing system consisting of a load cell (LTS-50GA and 10GA; KYOWA), a linear stage (M-111.1 DG; PI), a charge-coupled device camera (F-145Bs IRF; Marlin), and a water batch. The thickness of PEDOT:PSS hydrogels in the dried state and hydrated state was measured using the noncontact 3D surface profiler (NANO View-E1000; Nanosystem).

### Electrical characterization

The sheet resistance (*R*) of PEDOT:PSS hydrogels in the dried state and hydrated state was measured using a four-point probe (2400; Keithley). Using the thickness (*t*) data acquired earlier, the electrical conductivity (σ) was calculated with the following equation σ = 1/(*R* ∙ *t*)

For electrical resistance change measurement under strain, PEDOT:PSS hydrogels with dimensions of 10 mm × 10 mm (10 mm in length and 10 mm in width) were fabricated on a stretchable SBS substrate. A laboratory-made automated cyclic motion controller was used for stretching cycles.

### Electrochemical characterization

CV and EIS were conducted using a potentiostat (VersaSTAT 3; Princeton Applied Research). A platinum plate electrode was used as the counter electrode, and a Ag/AgCl (3 M KCl) electrode was used as the reference electrode. All samples were soaked in PBS for 1 hour before measurements. The cyclic voltammogram was recorded (versus Ag/AgCl reference electrode from −0.6 to 0.6 V). The frequency of EIS ranged between 1 and 10 kHz with a potential bias of 10 mV. For electrochemical measurements under strain, all PEDOT:PSS hydrogels were clamped in a laboratory-made stretch station and stretched up to 30% during CV and EIS. The charge injection capacity was performed by exerting cathodal first, biphasic, charge-balanced current pulse through chronoamperometry (−0.6 V versus Ag/AgCl for 10 ms and 0.6 V versus Ag/AgCl for 10 ms) using an electrochemical workstation (VersaSTAT 3; Princeton Applied Research).

### Stability characterization in a physiological environment

Aqueous stability in a physiological environment was tested by storing the PEDOT:PSS hydrogels in vials with PBS for 6 months. CV and EIS were performed after 6 months of storage.

### Mouse husbandry

Twelve-five–week–old ICR mice were purchased from OrientBio (Seongnam, South Korea) and randomly divided into three group (four mice per group). All experiments involving mice were performed with the approval of Konkuk University Institutional Animal Care and Use Committee (KU21150). All animals were maintained in a 12-hour light/12-hour dark cycle at 23° ± 1°C and 50 ± 10% relative humidity with free access to food and water.

### In vitro biocompatibility

L929 mouse fibroblast cells (KCLB, Seoul, South Korea) were cultured in Dulbecco’s modified Eagle’s medium (DMEM) (11885-084; Thermo Fisher Scientific, MA, USA) containing 10% fetal bovine serum (F2442; Sigma-Aldrich, MO, USA) and Anti-Anti (15240-062; Thermo Fisher Scientific) at 37°C with 5% CO_2_. PEDOT:PSS hydrogels with 1 wt % GOPS and 13 volume % DMSO additives were prepared by coating them on a TPU substrate with 1 hour of thermal annealing on a hot plate (130°C). All samples were detoxified with water for 1 hour after the annealing procedure and immediately used for in vitro toxicity evaluation. After cutting the hydrogels to a size corresponding to 10% of the surface of the cell culture plate and attaching it to the bottom, cells were seeded at a density of 50,000 cells/cm^2^ into a six-well culture plate (3516; Costar, MA, USA).

After 24 hours of incubation, bright-field images were obtained using the Nikon Eclipse TS100 microscope (Nikon, Japan) to analyze cell morphology changes. In addition, live and dead assay was performed using the LIVE/DEAD Viability/Cytotoxicity Kit (L3224; Invitrogen, CA, USA). In brief, 4 μM ethidium homodimer-1 and 2 μM calcein were added for 1 hour, and live (green) or dead (red) cells were observed under a Nikon Eclipse Ti fluorescence microscope (Nikon Instruments Inc., NY, USA). For cell viability quantification, the cells (three wells for each sample) were incubated for 1 hour at 37°C with MTT (M6494, Thermo Fisher Scientific) solution diluted to 0.5 mg/ml using PBS. After incubation, MTT solution was removed, and DMSO was used to dissolve MTT formazan. The absorbance was read at an optical density (OD) at 540 nm using the Epoch Microplate Spectrometer (BioTek Instruments Inc., VT, USA) and normalized to the control (unpaired, two-tailed Student’s *t* test).

### Histological analysis immunostaining

All four mice were used to analyze the effect of chronic implantation. Four weeks after implantation, the mice were euthanized with CO_2_ gas. The left thighs containing electrodes were sampled and fixed with 4% paraformaldehyde for 24 hours. Samples were sequentially transferred to a 15 and 30% sucrose solution overnight, followed by transfer to Optimal Cutting Temperature compound (OCT) (Sakura Finetek, CA, USA) and freezing at −70°C. The frozen samples were sectioned into 10-μm slices using the Microm HM525 NX Cryostat (Thermo Fisher Scientific, MA, USA). Slices were washed with distilled water and stained with hematoxylin and eosin (H&E) for histological analysis.

For immunofluorescence staining, the cryosectioned samples were fixed using absolute ethanol at −20°C for 10 min, washed with PBS, and preincubated for 30 min using a blocking reagent (1% bovine serum albumin). Subsequently, anti-neurofilament (801601; BioLegend, CA, USA) and anti-ED1 (ab125212; Abcam, MA, USA) were used as primary antibodies and incubated at 4°C overnight, followed by incubation with secondary antibodies for 2 hours. After washing with PBS, nuclei were stained using Vectashield mounting medium with 4′,6-diamidino-2-phenylindole (DAPI; Vector Laboratories, CA, USA), and the samples were photographed and merged using a Nikon Eclipse Ti fluorescence microscope (Nikon Instruments Inc.) and NIS-Elements software (Nikon). The fluorescence intensity was measured using ImageJ software (National Institutes of Health, Bethesda, MD, USA) and normalized with the mean value of the control (unpaired, two-tailed Student’s *t* test).

### Neural signal recording

Mice were euthanized with CO_2_ for neural recording. Brain slices (400 μm thick) containing the hippocampus were prepared using the Leica VT 1000M vibratome (Leica, Nussloch, Germany) with ice-cold artificial cerebrospinal fluid (CSF) (1.25 mM NaH_2_PO_4_, 26 mM NaHCO_3_, 125 mM NaCl, 2.5 mM KCl, 10 mM glucose, 2 mM CaCl_2_, and 1 mM MgCl_2_) bubbled with 95% O_2_/5% CO_2_ mixed gas. After dissection, brain slices were recovered in a 35°C water bath for 45 min. The CA3 region of the brain hippocampus was placed at the center of PA 10 electrodes and gently pressed for better contact. Then, the three electrodes of the recording device were connected to a ground pin, reference pin, and recording terminal of the laboratory-made signal reading circuit, respectively. For neuronal excitation, 20 μM bicuculline was added. To prevent surrounding electromagnetic waves from affecting the accuracy of LFP recordings, the whole setup was placed in a Faraday cage.

### Electrical stimulation and implantation of PA 10

All procedures involving electrode implantation and stimulation of the mouse sciatic nerve were performed with the approval of Konkuk University Institutional Animal Care and Use Committee (KU21150). Surgical procedures were conducted according to previous methods with some modifications. Mice were anesthetized with 2% isoflurane in balanced oxygen. Under sterile conditions, a 2-cm skin incision was made on the left thigh, and the sciatic nerve was exposed by separating the femoral muscles. Autoclaved 45° angled forceps were inserted below for sciatic nerve elevation, and stimulation devices made using PA 10 electrodes or rigid Au electrodes were placed under the nerve. The devices were wrapped around the sciatic nerve and slightly pulled for better contact at the interface. For the leg movement experiment, the sciatic nerve was stimulated with biphasic sinusoidal voltage pulses from a function waveform generator (DG1022; RIGOL Technologies). The movement of the ankle joint in response to the stimulation was measured for each device with increasing amplitude or frequency. After stimulation, the muscles and skin were closed with surgical sutures. The animals were observed for 2 hours after recovery and returned to their own cages. To examine the effects of chronic implantation, the animals were kept for 4 weeks after surgery. For behavior analysis, a stick diagram of the hindlimb was plotted on the basis of a video, which shows the mice walking along a 5 cm–by–40 cm track.

## Supplementary Materials

### This PDF file includes:

Supplementary Text Figs. S1 to S20 Tables S1 and S2 References

## Notes

View/request a protocol for this paper from *Bio-protocol*.

## References

[1] H. Yuk, B. Lu, X. Zhao, Hydrogel bioelectronics. Chem. Soc. Rev. 48, 1642–1667 (2019). 30474663 10.1039/c8cs00595h

[2] X. Fan, W. Nie, H. Tsai, N. Wang, H. Huang, Y. Cheng, R. Wen, L. Ma, F. Yan, Y. Xia, PEDOT:PSS for flexible and stretchable electronics: Modifications, strategies, and applications. Adv. Sci. 6, 1900813 (2019). 10.1002/advs.201900813 PMC6774040 31592415

[3] N. Kim, S. Lienemann, I. Petsagkourakis, D. A. Mengistie, S. Kee, T. Ederth, V. Gueskine, P. Leclere, R. Lazzaroni, X. Crispin, K. Tybrandt, Elastic conducting polymer composites in thermoelectric modules. Nat. Commun. 11, 1424 (2020). 32188853 10.1038/s41467-020-15135-w PMC7080746

[4] Y. Liu, J. Liu, S. Chen, T. Lei, Y. Kim, S. Niu, H. Wang, X. Wang, A. M. Foudeh, J. B. Tok, Z. Bao, Soft and elastic hydrogel-based microelectronics for localized low-voltage neuromodulation. Nat. Biomed. Eng. 3, 58–68 (2019). 30932073 10.1038/s41551-018-0335-6

[5] Y. Liu, J. Li, S. Song, J. Kang, Y. Tsao, S. Chen, V. Mottini, K. McConnell, W. Xu, Y. Zheng, J. B.-H. Tok, P. M. George, Z. Bao, Morphing electronics enable neuromodulation in growing tissue. Nat. Biotechnol. 38, 1031–1036 (2020). 32313193 10.1038/s41587-020-0495-2 PMC7805559

[6] Z. Aqrawe, J. Montgomery, J. T. Sejdic, D. Svirskis, Conducting polymers for neuronal microelectrode array recording and stimulation. Sens. Actuators B Chem. 257, 753–765 (2018).

[7] M. Ganji, E. Kaestner, J. Hermiz, N. Rogers, A. Tanaka, D. Cleary, S. H. Lee, J. Snider, M. Halgren, G. R. Cosgrove, B. S. Carter, D. Barba, I. Uguz, G. G. Malliaras, S. S. Cash, V. Gilja, E. Halgren, S. A. Dayeh, Development and translation of PEDOT:PSS microelectrodes for intraoperative monitoring. Adv. Funct. Mater. 28, 1700232 (2018).

[8] U. Lang, N. Naujoks, J. Dual, Mechanical characterization of PEDOT:PSS thin films. Synth. Met. 159, 473–479 (2009).

[9] B. Yao, H. Wang, Q. Zhou, M. Wu, M. Zhang, C. Li, G. Shi, Ultrahigh-conductivity polymer hydrogels with arbitrary structures. Adv. Mater. 29, 1700974 (2017). 10.1002/adma.201700974 28513994

[10] V. R. Feig, H. Tran, M. Lee, Z. Bao, Mechanically tunable conductive interpenetrating network hydrogels that mimic the elastic moduli of biological tissue. Nat. Commun. 9, 2040 (2018). 30013027 10.1038/s41467-018-05222-4 PMC6048132

[11] V. R. Feig, H. Tran, M. Lee, K. Liu, Z. Huang, L. Beker, D. G. Mackanic, Z. Bao, An electrochemical gelation method for patterning conductive PEDOT:PSS hydrogels. Adv. Mater. 31, 1902869 (2019). 10.1002/adma.201902869 31414520

[12] S. Zhang, Y. Chen, H. Liu, Z. Wang, H. Ling, C. Wang, J. Ni, B. C. Saltik, X. Wang, X. Meng, H. J. Kim, A. Baidya, S. Ahadian, N. Ashammakhi, M. R. Dokmeci, J. T. Sejdic, A. Khademhosseini, Room-temperature-formed PEDOT:PSS hydrogels enable injectable, soft, and healable organic bioelectronics. Adv. Mater. 32, 1904752 (2020). 10.1002/adma.201904752 PMC6946856 31657081

[13] B. Lu, H. Yuk, S. Lin, N. Jian, K. Qu, J. Xu, X. Zhao, Pure PEDOT:PSS hydrogels. Nat. Commun. 10, 1043 (2019). 30837483 10.1038/s41467-019-09003-5 PMC6401010

[14] H. Yuk, B. Lu, S. Lin, K. Qu, J. Xu, J. Luo, X. Zhao, 3D printing of conducting polymers. Nat. Commun. 11, 1604 (2020). 32231216 10.1038/s41467-020-15316-7 PMC7105462

[15] M. S. Mahajan, D. M. Marathe, S. S. Ghosh, V. Ganesan, J. V. Sali, Changes in in-plane electrical conductivity of PEDOT:PSS thin films due to electric field induced dipolar reorientation. RSC Adv. 5, 86393–86401 (2015).

[16] K. Lim, J. Kang, S. Jung, S. Lee, J. Park, D. G. Kim, Y. C. Kang, Improving electrical conductivity of PEDOT:PSS with phase separation by applying electric fields. Bull. Korean Chem. Soc. 39, 469–476 (2018).

[17] N. Chaturvedi, F. Alam, S. K. Swami, V. Dutta, Effect of electric field on the spray deposited poly (3,4-ethylenedioxythiophene): Poly(styrenesulfonate) layer and its use in organic solar cell. J. Appl. Phys. 114, 184501 (2013).

[18] L. D. Garma, L. M. Ferrari, P. Scognamiglio, F. Greco, F. Santoro, Inkjet-printed PEDOT:PSS multi-electrode arrays for low-cost in vitro electrophysiology. Lab Chip 19, 3776–3786 (2019). 31616896 10.1039/c9lc00636b

[19] M. Y. Teo, N. RaviChandran, N. Kim, S. Kee, L. Stuart, K. C. Aw, J. Stringer, Direct patterning of highly conductive PEDOT:PSS/ionic liquid hydrogel via microreactive inkjet printing. ACS Appl. Mater. Interfaces 11, 37069–37076 (2019). 31533420 10.1021/acsami.9b12069

[20] D. N. Heo, S. J. Lee, R. Timsina, X. Qiu, N. J. Castro, L. G. Zhang, Development of 3D printable conductive hydrogel with crystallized PEDOT: PSS for neural tissue engineering. Mater. Sci. Eng. C 99, 582–590 (2019). 10.1016/j.msec.2019.02.008 30889733

[21] C. Yun, J. W. Han, S. Kim, D. C. Lim, H. Jung, S. H. Lee, J. W. Jang, S. Yoo, K. Leo, Y. H. Kim, Generating semi-metallic conductivity in polymers by laser-driven nanostructural reorganization. Mater. Horiz. 6, 2143–2151 (2019).

[22] S. Chae, K. H. Jo, S. W. Lee, H. S. Keum, H. J. Kim, J. Choi, H. H. Lee, Selective chain alignment of conducting polymer blend films by an ultrafast laser. Macromol. Chem. Phys. 217, 537–542 (2016).

[23] C. Qin, X. Zhang, W. He, G. Zhang, R. Chen, Y. Gao, L. Xiao, S. Jia, Continuous-wave laser-induced welding and giant photoluminescence enhancement of Au nanospheres. Opt. Express 27, 2886–2898 (2019). 30732319 10.1364/OE.27.002886

[24] I. Song, N. Y. Park, G. S. Jeong, J. H. Kang, J. H. Seo, J.-Y. Choi, Conductive channel formation for enhanced electrical conductivity of PEDOT:PSS with high work-function. Appl. Surf. Sci. 529, 147176 (2020).

[25] S. M. Kim, C. H. Kim, Y. Kim, N. Kim, W. J. Lee, E. H. Lee, D. Kim, S. Park, K. Lee, J. Rivnay, M. H. Yoon, Influence of PEDOT:PSS crystallinity and composition on electrochemical transistor performance and long-term stability. Nat. Commun. 9, 3858 (2018). 30242224 10.1038/s41467-018-06084-6 PMC6155079

[26] A. Hu, L. Tan, X. Hu, L. Hu, Q. Ai, X. Meng, L. Chen, Y. Chen, Crystallization and conformation engineering of solution-processed polymer transparent electrodes with high conductivity. J. Mater. Chem. C. 5, 382–389 (2017).

[27] J. Nevrela, M. Micjan, M. Novota, S. Kovacova, M. Pavuk, P. Juhasz, J. Kovac, J. Jakabovic, M. Weis, Secondary doping in poly(3,4-ethylenedioxythiophene):Poly(4-styrenesulfonate) thin films. J Polym Sci B 53, 1139–1146 (2015).

[28] Q. Li, J. Yang, S. Chen, J. Zou, W. Xie, X. Zeng, Highly conductive PEDOT: PSS transparent hole transporting layer with solvent treatment for high performance silicon/organic hybrid solar cells. Nanoscale Res. Lett. 12, 506 (2017). 28836136 10.1186/s11671-017-2276-5 PMC6890909

[29] L. Qian, H. Zhao, Nanoindentation of soft biological materials. Micromachines. 9, 654 (2018). 30544918 10.3390/mi9120654 PMC6316095

[30] J. H. Kim, A. Nizami, Y. Hwangbo, B. Jang, H. J. Lee, C. S. Woo, S. Hyun, T. S. Kim, Tensile testing of ultra-thin films on water surface. Nat. Commun. 4, 2520 (2013). 24084684 10.1038/ncomms3520

[31] R. Akhtar, M. J. Sherratt, J. K. Cruickshank, B. Derby, Characterizing the elastic properties of tissues. Mater. Today 14, 96–105 (2011). 10.1016/S1369-7021(11)70059-1 PMC3378034 22723736

[32] K. Feron, R. Lim, C. Sherwood, A. Keynes, A. Brichta, P. C. Dastoor, Organic bioelectronics: Materials and biocompatibility. Int. J. Mol. Sci. 19, 2382 (2018). 30104515 10.3390/ijms19082382 PMC6121695

[33] L. D. Medinaceli, W. J. Freed, R. J. Wyatt, An index of the functional condition of rat sciatic nerve based on measurements made from walking tracks. Exp. Neurol. 77, 634–643 (1982). 7117467 10.1016/0014-4886(82)90234-5

[34] M. J. Piesla, L. Leventhal, B. W. Strassle, J. E. Harrison, T. A. Cummons, P. Lu, G. T. Whiteside, Abnormal gait, due to inflammation but not nerve injury, reflects enhanced nociception in preclinical pain models. Brain Res. 1295, 89–98 (2009). 19651113 10.1016/j.brainres.2009.07.091

[35] A. G. Sulser, J. Wang, B. N. Queenan, M. Avoli, S. Vicini, R. Dzakpasu, Hippocampal neuron firing and local field potentials in the in vitro 4-aminopyridine epilepsy model. J. Neurophysiol. 108, 2568–2580 (2012). 22972961 10.1152/jn.00363.2012 PMC3545168

[36] V. Viswam, M. E. J. Obien, F. Franke, U. Frey, A. Hierlemann, Optimal electrode size for multi-scale extracellular-potential recording from neuronal assemblies. Front. Neurosci. 13, 385 (2019). 31105515 10.3389/fnins.2019.00385 PMC6498989

[37] A. O. Rosario, H. Adeli, Brain-computer interface technologies: From signal to action. Rev. Neurosci. 24, 537–552 (2013). 24077619 10.1515/revneuro-2013-0032

[38] A. H. Bozer, M. L. Uhelski, A. L. Li, Extrapolating meaning from local field potential recordings. J. Integr. Neurosci. 16, 107–126 (2017). 28891502 10.3233/JIN-170011

[39] I. Khalilov, V. Dzhala, Y. B. Ari, R. Khazipov, Dual role of GABA in the neonatal rat hippocampus. Dev. Neurosci. 21, 310–319 (1999). 10575254 10.1159/000017380

[40] R. Stoop, F. Conquet, B. Zuber, L. L. Voronin, E. Pralong, Activation of metabotropic glutamate 5 and NMDA receptors underlies the induction of persistent bursting and associated long-lasting changes in CA3 recurrent connections. J. Neurosci. 23, 5634–5644 (2003). 12843266 10.1523/JNEUROSCI.23-13-05634.2003 PMC6741217

[41] M. Solazzo, K. Krukiewicz, A. Zhussupbekova, K. Fleischer, M. J. Biggs, M. G. Monaghan, PEDOT: PSS interfaces stabilised using a PEGylated crosslinker yield improved conductivity and biocompatibility. J. Mater. Chem. B 7, 4811–4820 (2019). 31389966 10.1039/c9tb01028a

