# Supplementary material for: Digital selective transformation and patterning of highly conductive hydrogel bioelectronics by laser-induced phase separation
Source: Sci Adv. 2022 Jun 8;8(23):eabo3209. doi: 10.1126/sciadv.abo3209 (PMC9177068; doi:10.1126/sciadv.abo3209)
Supplement: Supplementary file 1 — Supplementary Text Figs. S1 to S20 Tables S1 and S2 References [file sciadv.abo3209_sm.pdf]

Science Advances
AAAS

# Supplementary Materials for

## Digital selective transformation and patterning of highly conductive hydrogel bioelectronics by laser-induced phase separation

Daeyeon Won *et al.*

Corresponding author: Seung Hwan Ko, maxko@snu.ac.kr; C-Yoon Kim, vivavet@konkuk.ac.kr

*Sci. Adv.* **8**, eabo3209 (2022)
DOI: 10.1126/sciadv.abo3209

**The PDF file includes:**

Supplementary Text
Figs. S1 to S20
Tables S1 and S2
Legends for movies S1 to S4
References

**Other Supplementary Material for this manuscript includes the following:**

Movies S1 to S4

## Supplementary Text

### Estimation of LIPSP to the depth direction.

Since it is difficult to measure an accurate resolution of LIPSP in the depth direction, we estimated the relative intensity of the laser in the target depth whether it can provide sufficient threshold intensity for the phase separation using fundamental Gaussian beam modeling. To find out the resolution in the depth direction where the phase separation by the laser occurs, it is necessary to consider the change in the collimation and beam intensity in the depth direction. First, the laser beam used in the LIPSP is set to the combination of optics described in **fig. S9A**, and has an output beam diameter of 7 µm from the telecentric f-theta lens. On the other hand, the area where phase separation occurs by the laser is at the level of 6 µm. Therefore, the relative intensity of the threshold point of LIPSP (i.e. at r = 3 µm) to the peak intensity (i.e., at r = 0 µm) of the laser beam was calculated. (**fig. S9B**). The intensity of a Gaussian beam can be calculated by the following equation.

$$I(r) = \frac{2P}{\pi {w_0}^2} exp(\frac{-2r^2}{{w_0}^2})$$

$$\frac{I(w_0, \ \ r = 3)}{I(w_0, \ \ r = 0)} = 0.23$$

Where I(r) is the intensity of the Gaussian laser beam as a function of radial distance from the center of the beam, P is laser power, $w_0$ is beam waist (i.e., half of beam diameter, 3.5 µm), and, r is the radial distance from the center of the beam.

Second, we calculated the Rayleigh length ($z_R$), the depth at which the Gaussian laser beam maintains the collimation of the degree of beam radius increase by square root 2 (**fig. S9C**).

$$z_R = \frac{\pi w_0^2}{\lambda}$$

Where λ is the wavelength of the laser, and $w_0$ is a beam waist from the telecentric f-theta lens. The calculated $z_R$ value is 65.8 µm which is deep enough to treat thick film of PEDOT:PSS hydrogels. In order to confirm that the phase separation of PEDOT:PSS is sufficiently achieved at the depth of Rayleigh length, the relative intensity at $z_R$ was calculated. Before calculating the intensity, the beam radius of the Gaussian laser beam at $z_R$ has to be first found.

$$w(z) = w_0 \sqrt{1 + (\frac{z}{z_R})^2}$$

Where w(z) is the beam radius of the Gaussian laser beam at depth z from the focal plane. The intensity at an arbitrary depth and radial distance to the center of the laser beam can be found from the calculated w(z) value.

$$I(r) = \frac{2P}{\pi w(z)^2} exp(\frac{-2r^2}{w(z)^2})$$

$$\frac{I(w = w_R,\ r = 3\ )}{I(w_0,\ r = 0)} = 0.24 > 0.23 = \frac{I(w_0,\ \ r = 3\ )}{I(w_0,\ \ r = 0)}$$

The relative intensity of the Gaussian beam at the depth of $z_R$ and position of r = 3 μm was calculated as 0.24 which is higher than the threshold point of LIPSP.

The Gaussian beam profile was also plotted at the focal plane (i.e., z = 0 and w(z) = $w_0$) and at the Rayleigh length (i.e., z = $z_R$ and w(z) = $w_R$) (**fig. S9D**). The intensity of the Gaussian laser beam at $z_R$ and the position of r = 3 μm was revealed to be higher than that of the intensity of the beam at the focal plane (**fig. S9D, (i)**) which enables phase separation of PEDOT:PSS at the corresponding depth level.

The depth of the surface where we processed the PEDOT:PSS with a thickness of 10 μm was also studied. With a thickness of 10 μm, PEDOT:PSS hydrogels can secure sufficiently high electrochemical and electrical properties. In this case, the depth from the center of the PEDOT:PSS sample to the surface becomes 5 μm. It showed that there was little difference in the intensity between the center and the surface (**fig. S9D, (ii)**). Finally, we demonstrated that PEDOT:PSS with a thickness of about 10 μm was uniformly processed by the LIPSP (**fig. S9E, F, and G**).

**Calculation of water contents of swollen PEDOT:PSS hydrogels**

To calculate the swelling ratio and water contents, the thickness of micropatterned PEDOT:PSS hydrogels in the fully dried state and swollen state were measured through a 3D surface profiler (NANO View-E1000, Korea). The samples were fully dried at room temperature for 1 day before measurement. After measuring the thickness of dried samples, they were immersed in DI water for 1h, then the swollen thicknesses were taken. As a result of the experiment, definite anisotropic swelling behaviors were observed (**fig. S14A**), thus lateral expansion rarely occurred. Therefore, only the change of thickness in z-direction was considered in the volume change of PEDOT:PSS hydrogels.

We modeled the PEDOT:PSS hydrogels as a cuboid and hypothesized that the volume change of the PEDOT:PSS hydrogel was solely due to water absorption (**fig. S15**). The volume of PEDOT:PSS in the dried state and the swollen state can be obtained simply by multiplying the lengths of the three sides. Where $x_1$, $y_1$, and $z_1$ are lengths in the dried state and $x_2$, $y_2$, and $z_2$ are in the swollen state.

$$V_{dried\ state} = x_1 * y_1 * z_1$$

$$V_{swollen\ state} = x_2 * y_2 * z_2$$

Since the lengths in the x- and y-axis does not change due to anisotropic swelling of PEDOT:PSS hydrogels, the swelling ratio and the change of volume due to water absorption are only dependent on thickness change in the z-direction.

$$Swelling\ ratio = \frac{V_{swollen\ state}}{V_{dried\ state}} = \frac{x_2 * y_2 * z_2}{x_1 * y_1 * z_1}$$

$$Water\ contents = \frac{V_{swollen\ state} - V_{dried\ state}}{V_{swollen\ state}} = \frac{x_2 * y_2 * z_2 - x_1 * y_1 * z_1}{x_2 * y_2 * z_2}$$

$$x_1 = x_2\ (anisotropic\ swelling)$$

$$y_1 = y_2\ (anisotropic\ swelling)$$

Therefore, the swelling ratio and water contents of PEDOT:PSS hydrogels can be obtained by simple calculation of measured thickness of PEDOT:PSS hydrogels as follows.

$$Swelling\ ratio = \frac{z_2}{z_1}$$

$$Water\ contents = \frac{z_2 - z_1}{z_2}$$

## Supplementary Figures and Tables

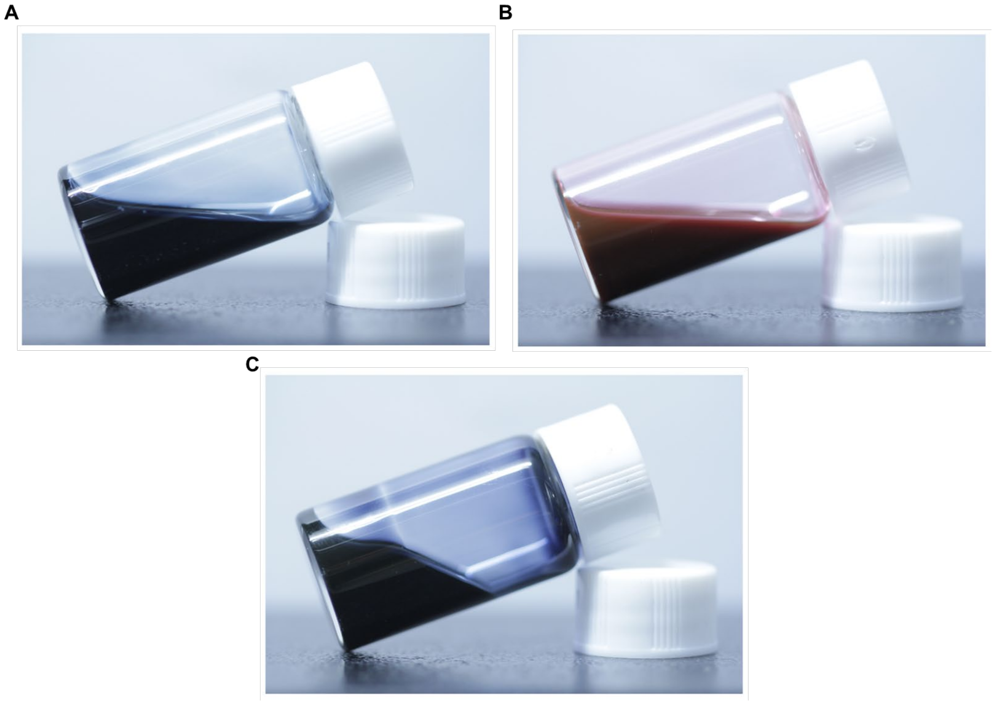


**Fig. S1. Preparation of PEDOT:PSS and AuNP composite inks (PA inks).**

(**A**) Pure PEDOT:PSS solution was blended with (**B**) AuNP inks in the range of 1-20 vol% of the (**C**) final PA inks (digital image shows PA 10 inks).

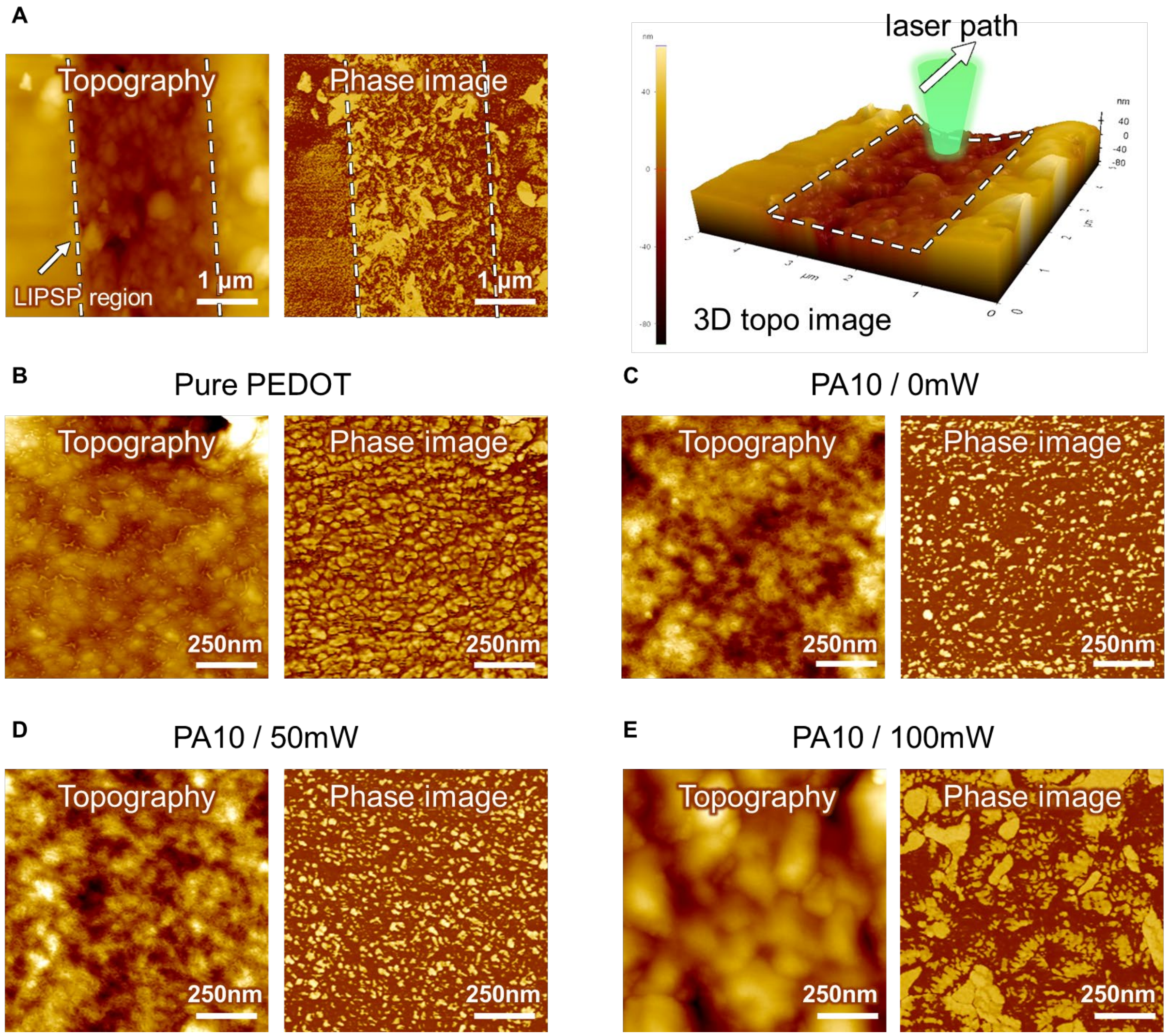


**Fig. S2. AFM analysis on laser-treated PEDOT:PSS.**

(**A**) Selectively redistributed phase of PEDOT:PSS by LIPSP (topography image on left and phase image on right). (**B**) Topography and phase image of Pure PEDOT:PSS without laser treatment. (**C**) Topography and phase image of PA 10 without laser treatment. (**D**) Topography and phase image of PA 10 treated by 50 mW of laser power. (**E**) Topography and phase image of PA 10 treated by 100 mW of laser power. All samples were scanned at 300 mm/s.

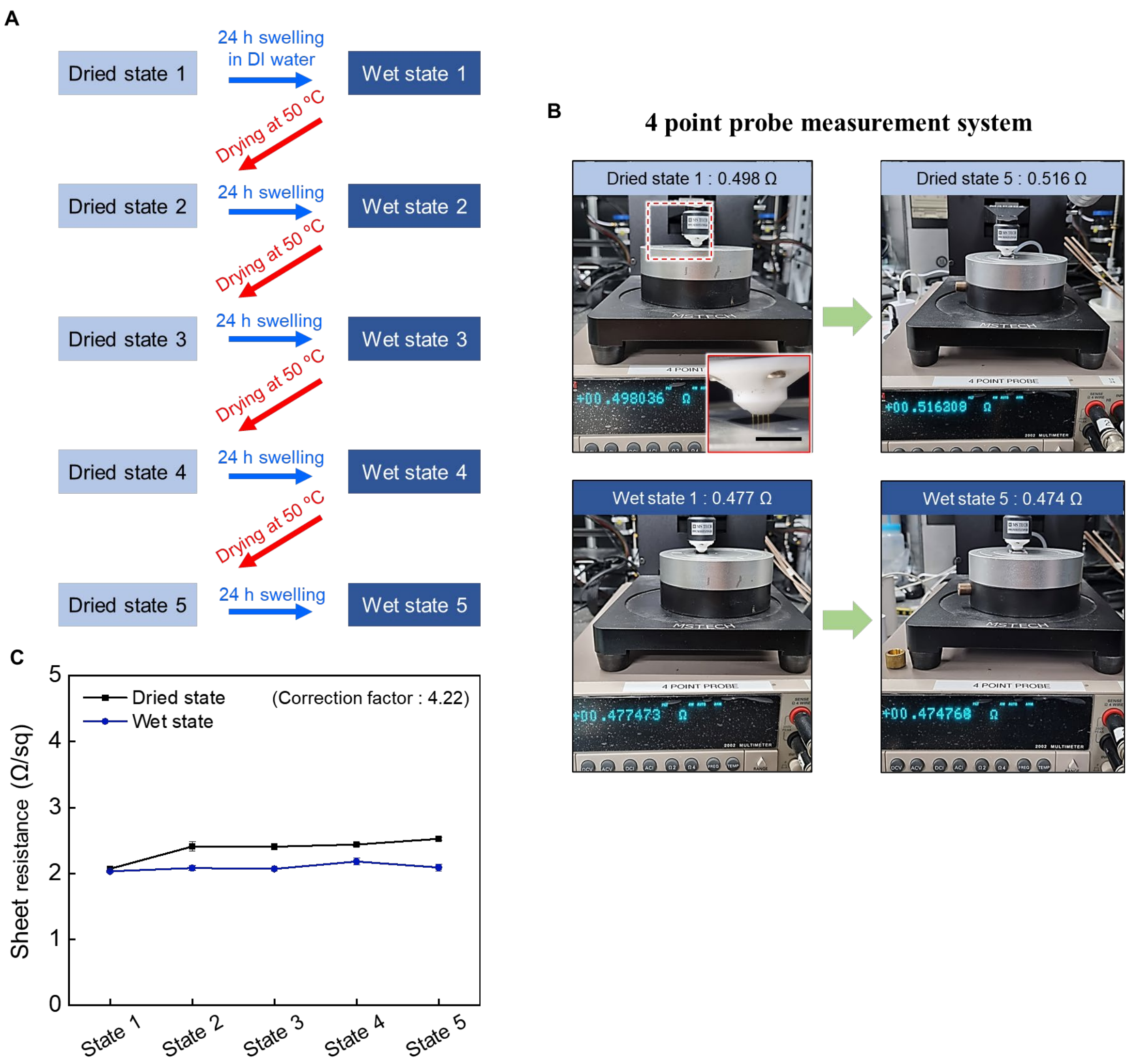


**Fig. S3. Electrical stability of PEDOT:PSS hydrogels during swelling-drying cycles.**

(**A**) Experiment flow of electrical stability test. (**B**) Digital images of the four-point probe measurement system. It showed no significant change of electrical resistance change of dried state and wet state. Scale bar in inset image is 10 mm. (**C**) Sheet resistance of PEDOT:PSS hydrogels in the dried and wet state during swelling-drying cycles. Values in (**C**) represent the mean and the error bars represent the standard deviation (n = 3).

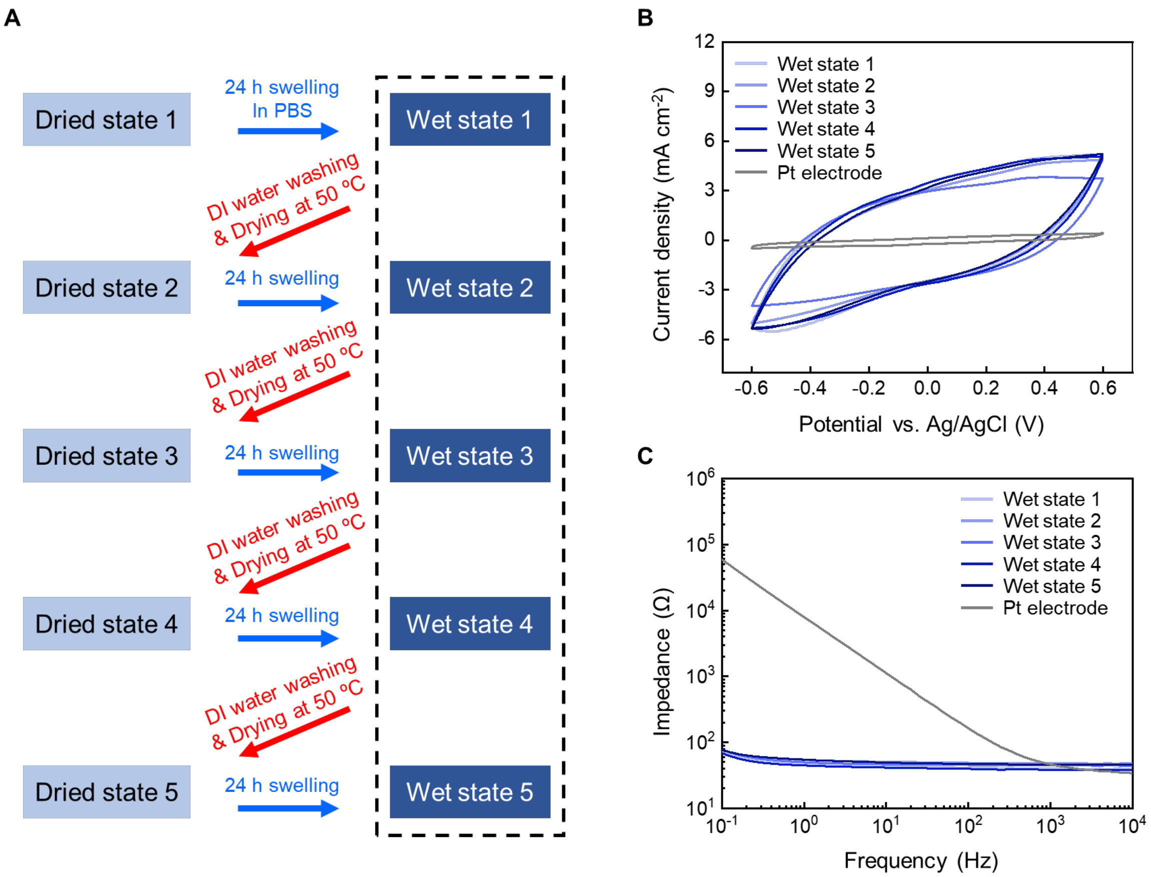


**Fig. S4. Electrochemical stability of PEDOT:PSS hydrogels during swelling-drying cycles.** (**A**) Experiment flow of electrochemical stability test. (**B**) Cyclic voltammetry curves of each wet state. (**C**) Electrochemical impedance spectroscopy of each wet state. It showed no significant change of electrochemical properties after multiple swelling-drying cycles.

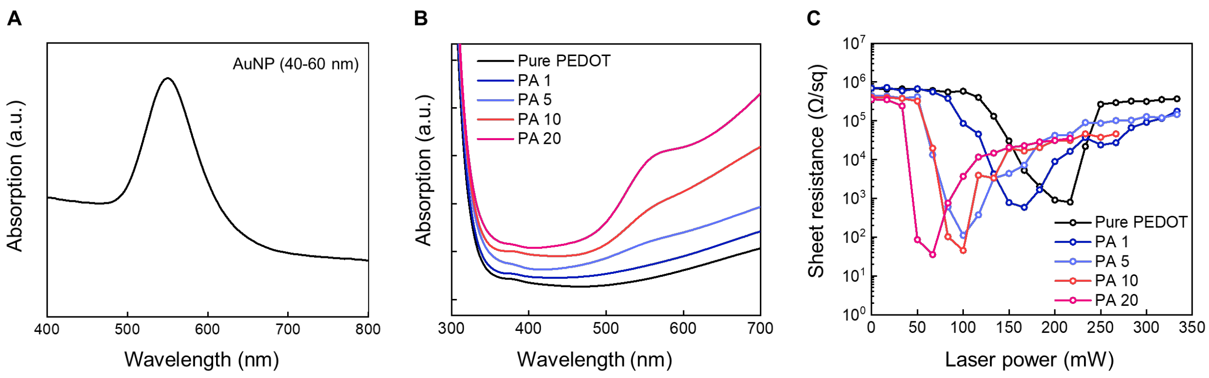


**Fig. S5. Effect of AuNP inks volume fraction on laser absorption and laser parameter.**

(**A**) UV-Vis spectroscopy of AuNP spin-coated on the glass substrate. (**B**) UV-Vis spectroscopy of PEDOT:PSS varying AuNP inks volume fraction. The absorption in the wavelength range between 500-600 nm was sufficiently increased by AuNP (Wavelength of laser = 532 nm). (**C**) The sheet resistance changes of PEDOT:PSS depending on laser parameters (scan speed = 300 mm/s). The optimum laser power for the highest electrical conductivity decreased with AuNP concentration.

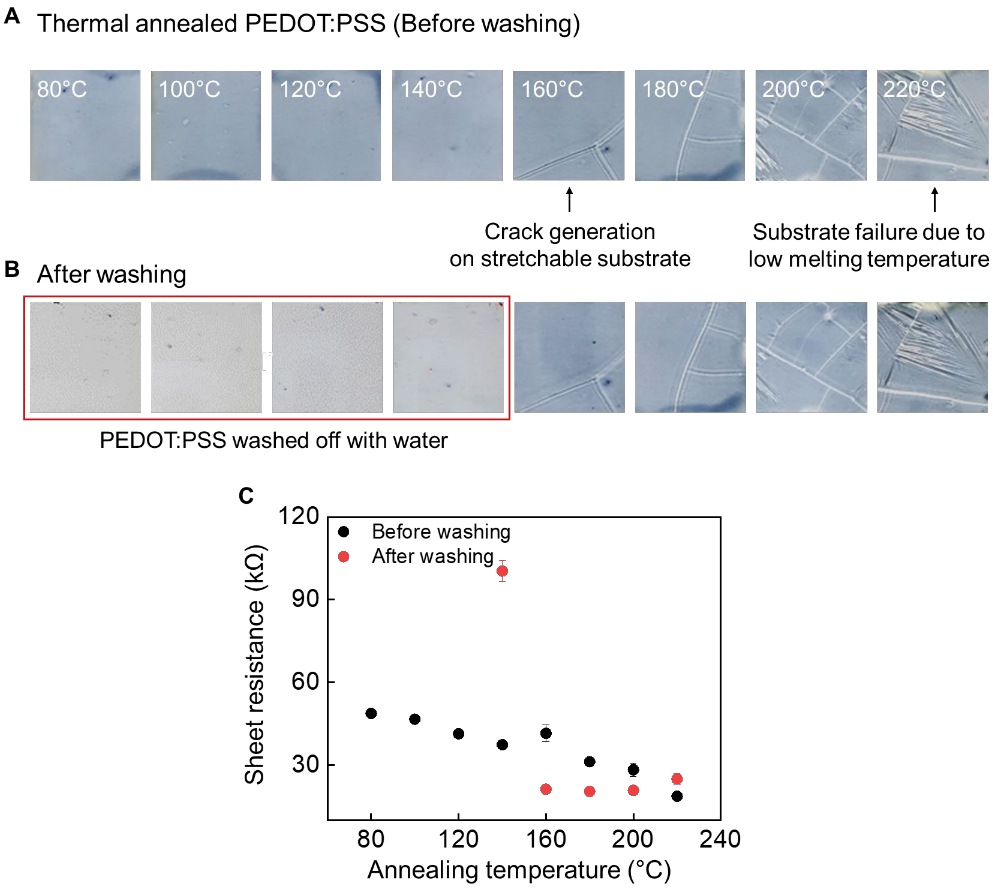


**Fig. S6. Bulk thermal annealing of PEDOT:PSS on the stretchable substrate (TPU).**

(**A**) The digital images of PEDOT:PSS samples annealed at different temperatures before washing with water. The destruction of the substrate starts from the annealing temperature of 160 °C. (**B**) The digital images of PEDOT:PSS samples after washing with water. The samples annealed under 140 °C were dissolved in water and delaminated from the substrate (red box). (**C**) The change of sheet resistance of PEDOT:PSS samples with varying annealing temperature. Values in (**C**) represent the mean and the error bars represent the standard deviation (n = 3).

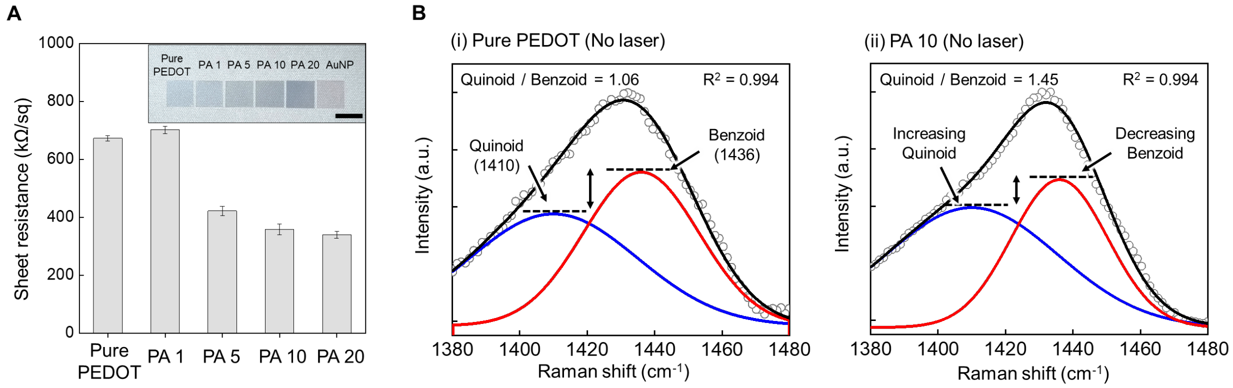


**Fig. S7. Electrical conductivity enhancement of PEDOT:PSS hydrogels by adding AuNP inks.**

(**A**) The decrease of sheet resistance of PEDOT:PSS with increasing volume fraction of AuNP inks. The inset image shows the color of spin-coated samples (scale bar is 10 mm). (**B**) Raman spectroscopy of PEDOT:PSS (i) before adding AuNP inks and (ii) after adding 10 vol% of AuNP inks. Values in (**A**) represent the mean and the error bars represent the standard deviation (n = 3).

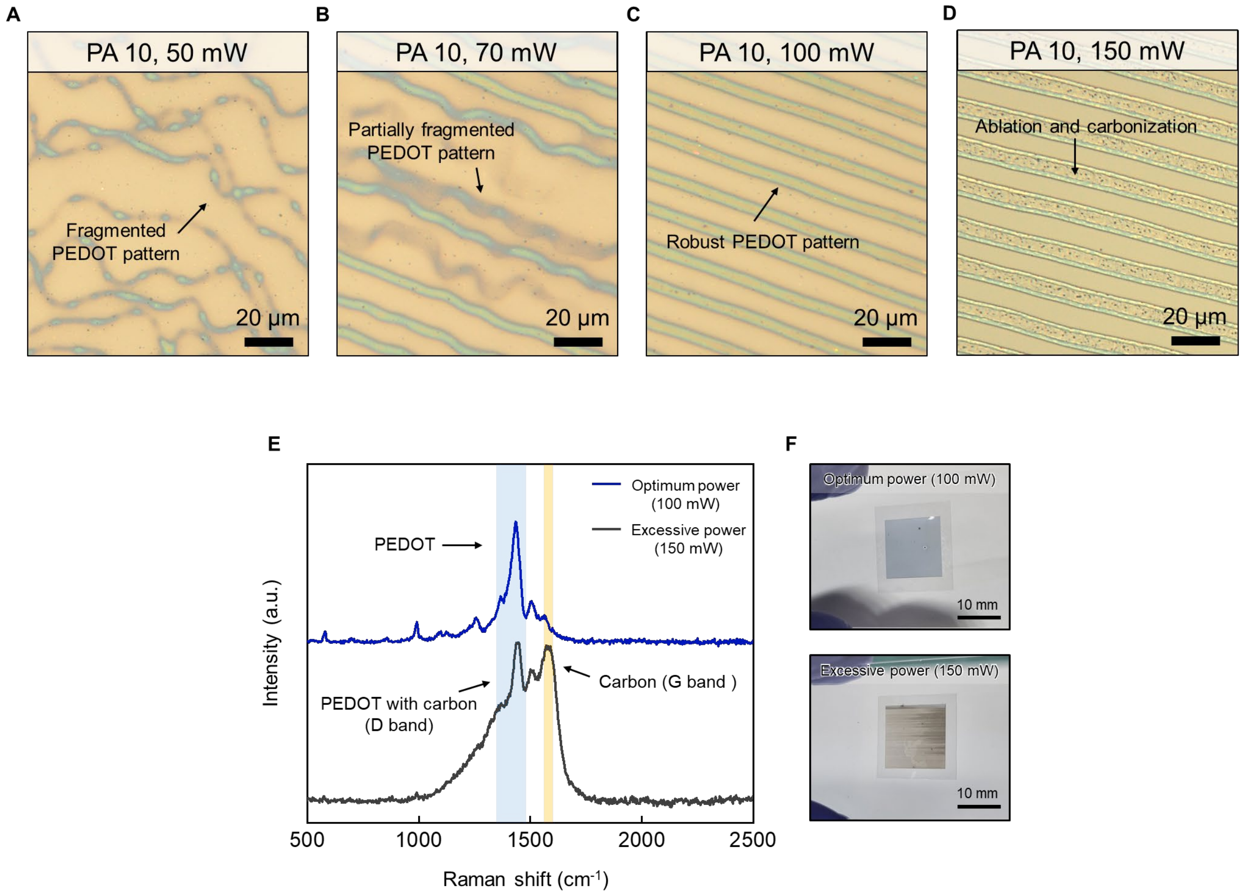


**Fig. S8. The patterning results of PEDOT:PSS hydrogels according to laser conditions.**
(**A**), (**B**) Low power condition. PEDOT:PSS was fragmented when immersed in water. (**C**) Optimum condition. The robust PEDOT:PSS hydrogel micropattern was formed. (**D**) High power condition. PEDOT:PSS hydrogels began to be carbonized and ablated. (**E**) Raman spectrum of PEDOT:PSS hydrogels under optimum condition and excessive high power condition. (**F**) Digital images of corresponding samples.

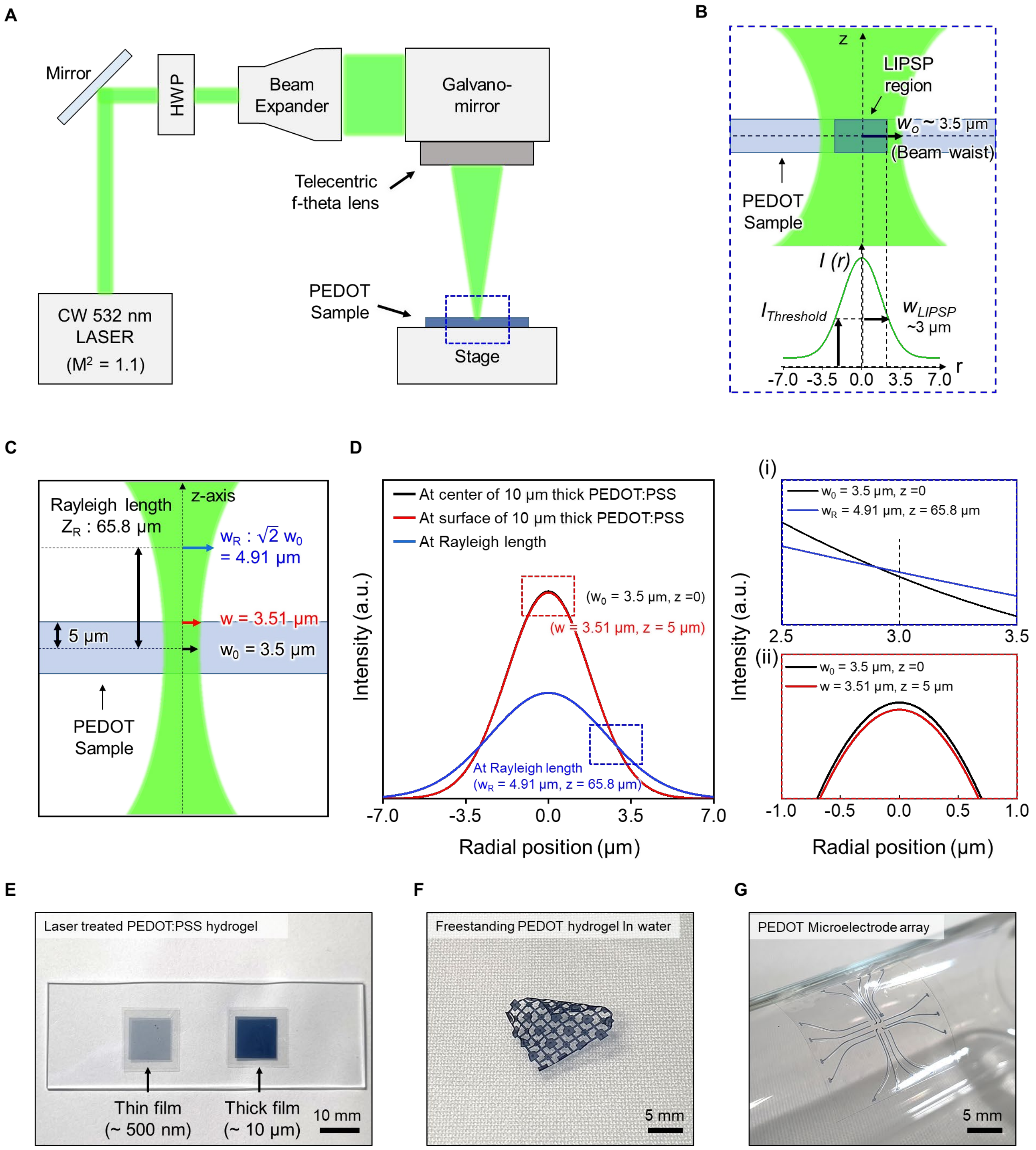


**Fig. S9. Laser optical system for LIPSP and depth resolution.**

(**A**) Laser optical system for the LIPSP. (**B**) Gaussian laser beam propagation and profile. (**C**) Schematic illustration of characteristic lengths of Gaussian laser beam. (**D**) Beam intensity profile of laser at different positions. (**E**) PEDOT:PSS hydrogels with the same geometrical area but different thickness, Both two samples were treated at the same focus. (**F**) Freestanding PEDOT:PSS hydrogels pattern floating in DI water. (**G**) Fabricated thick PEDOT microelectrode array on the stretchable substrate for the soft neural signal recording application.

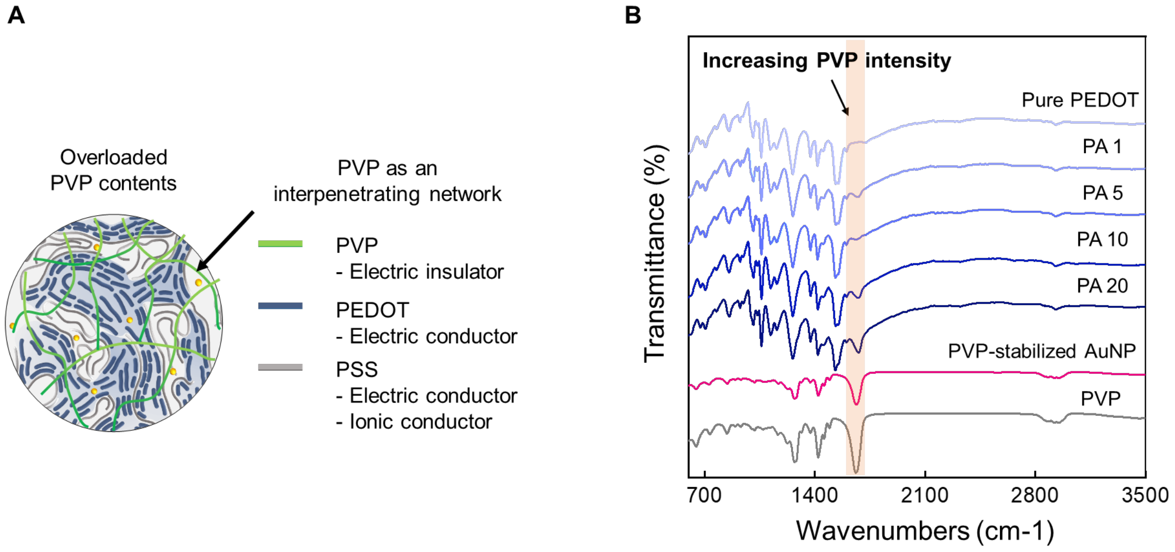


**Fig. S10. FT-IR Spectroscopy for PVP contents characterizations in PEDOT:PSS hydrogels according to the volume fraction of AuNP inks.**

(**A**) Schematic illustration of the role of PVP as an interpenetrating polymer network and electrical insulator for PEDOT:PSS hydrogels. (**B**) FT-IR Spectroscopy of PEDOT:PSS hydrogels according to the volume fraction of AuNP inks.

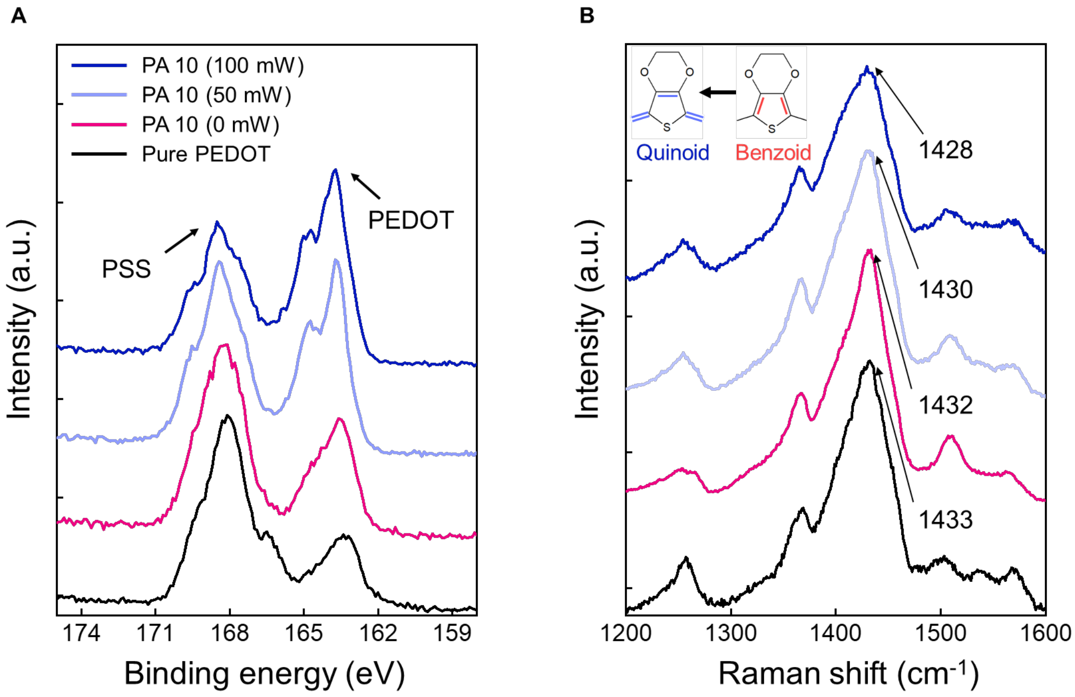


**Fig. S11. XPS and Raman spectroscopy of PEDOT:PSS (PA 10) with varying laser parameters.**

(**A**) XPS spectroscopy data with varying laser parameters. (**B**) Raman spectroscopy data with varying laser parameters. All samples were scanned at 300 mm/s.

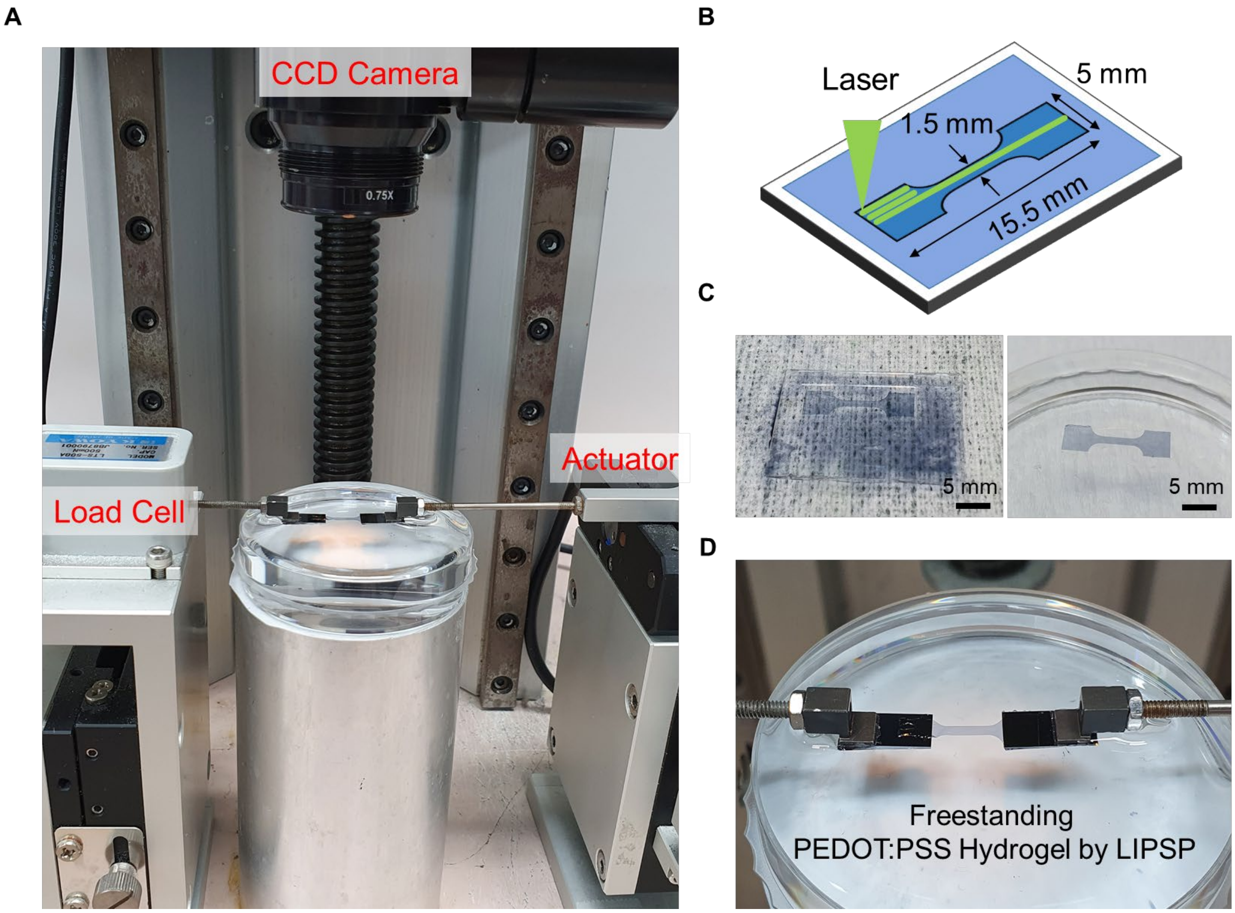


**Fig. S12. Experiment set up for tensile testing of PEDOT:PSS hydrogels.**

(**A**) Digital images of tensile testing set up. (**B**) The design of dog-bone shape specimen of PEDOT:PSS hydrogels. (**C**) Washing non-treated area by water. (**D**) Freestanding PEDOT:PSS hydrogel gripped by the gripper.

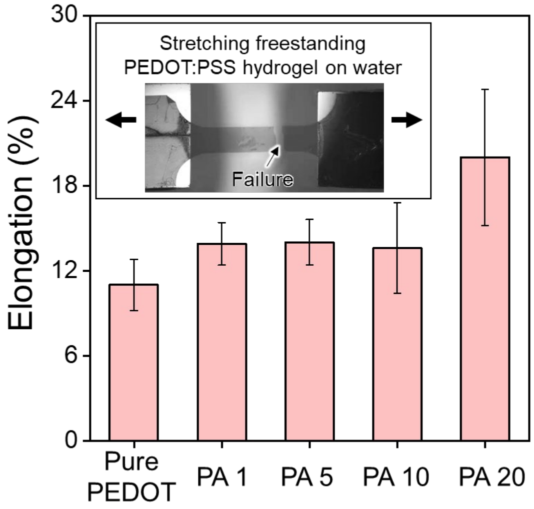


**Fig. S13. Elongation of PEDOT:PSS hydrogels from a tensile test in water.**

Inset shows an optical image of failure of PEDOT:PSS hydrogel in water. Values in figure represent the mean and the error bars represent the standard deviation (n = 3).

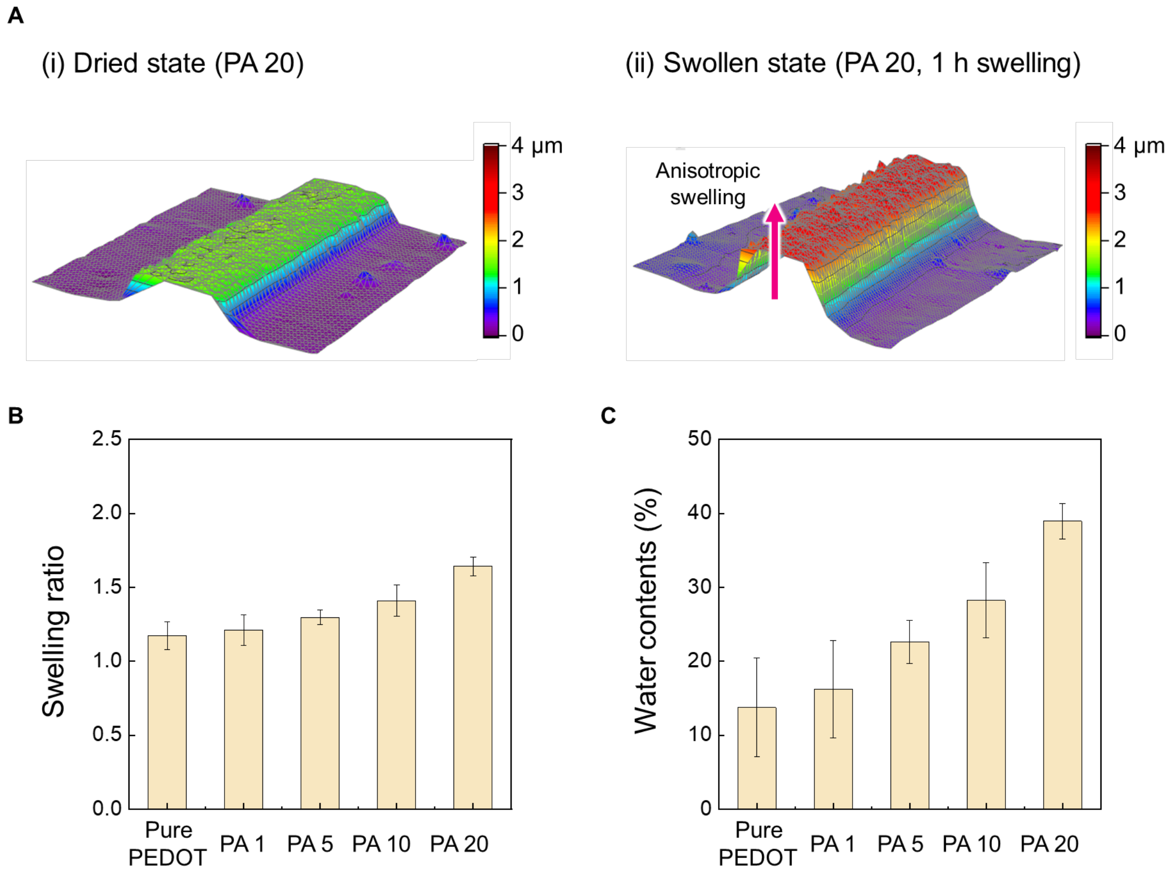


**Fig. S14. Anisotropic swelling behavior of PEDOT:PSS hydrogels.**

(**A**) 3D profile image of dried PA 20 and swollen PA 20. (**B**) The swelling ratio was calculated by dividing the thickness of the hydrated samples by that of the dried samples. (**C**) Water contents of the LIPSP hydrogels varying AuNP volume fraction. Values in (**B, C**) represent the mean and the error bars represent the standard deviation (n = 3).

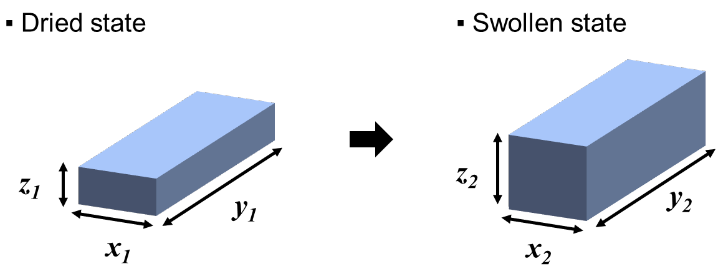


**Fig. S15. Calculation of water contents of swollen PEDOT:PSS hydrogels.**

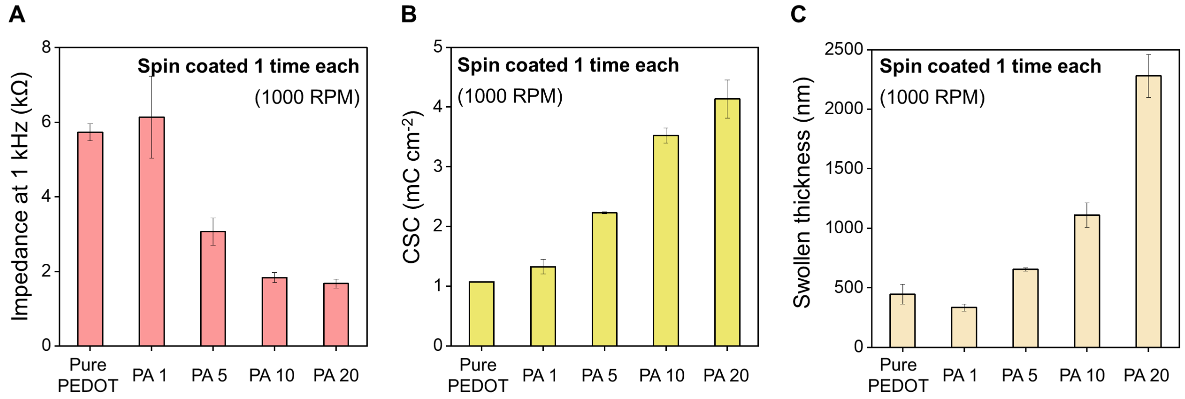


**Fig. S16. Electrochemical characterization of laser-treated PEDOT:PSS hydrogels depending on AuNP concentrations.**

(**A**) Impedance (Z) results of PEDOT:PSS hydrogels with 0.25 $mm^2$ of exposed area. (**B**) Charge storage capacity (CSC) results of PEDOT:PSS hydrogels with 0.25 $mm^2$ of exposed area. (**C**) The swollen thickness of PEDOT:PSS hydrogels. All samples were spin-coated on the substrate with 1000 rpm for 30 s. Values in (**A-C**) represent the mean and the error bars represent the standard deviation (n = 3).

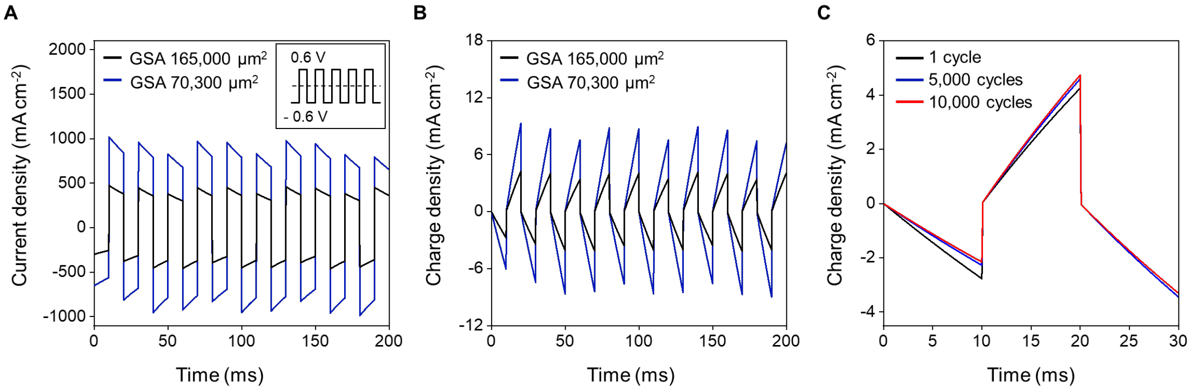


**Fig. S17. Charge injection capability of PEDOT:PSS hydrogels.**

(**A**) The current density of PEDOT:PSS hydrogels. (**B**) The charge density of PEDOT:PSS hydrogels. (**C**) Electrochemical stability during cyclic charge injection of PEDOT:PSS hydrogels. Since the CIC values are dependent on the geometric surface area (GSA) of electrode material, we tested two different electrodes of GSA for comparison. PA 10 electrode with GSA of 165,000 $\mu m^2$ showed the current density of 473 mA $cm^{-2}$ and 1,019 mA $cm^{-2}$ for GSA of 70,300 $\mu m^2$ which is much higher than conventional metallic electrodes such as platinum and gold. CIC was then calculated by integrating chronoamperometric curves, which showed 7.45 mC $cm^{-2}$ for GSA of 165,000 $\mu m^2$ and 16.17 mC $cm^{-2}$ for GSA of 70,300 $\mu m^2$.

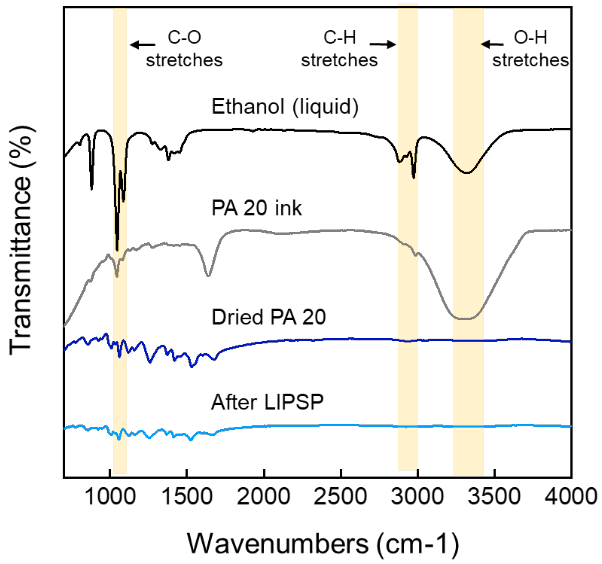


**Fig. S18. FT-IR spectroscopy for ethanol residue characterization.**

The C-O stretches, C-H stretches, and O-H stretches from pure ethanol and PA 20 ink were completely disappeared as we simply dried the PA inks at room temperature. PA 20 sample after laser process also showed no peak corresponding to ethanol.

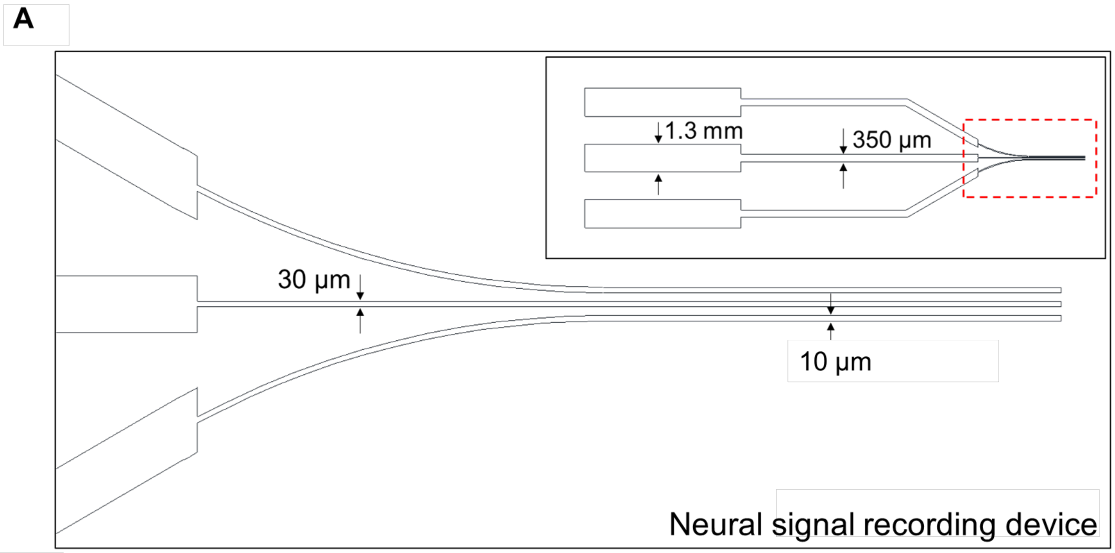


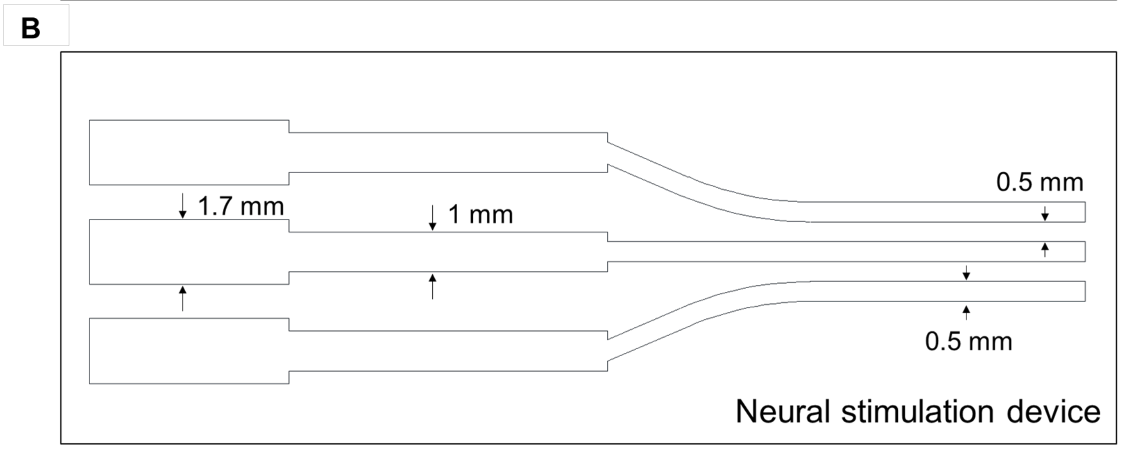


**Fig. S19. Design of bioelectronic devices.**
(**A**) Neural signal recording device, (**B**) Neural stimulation device.

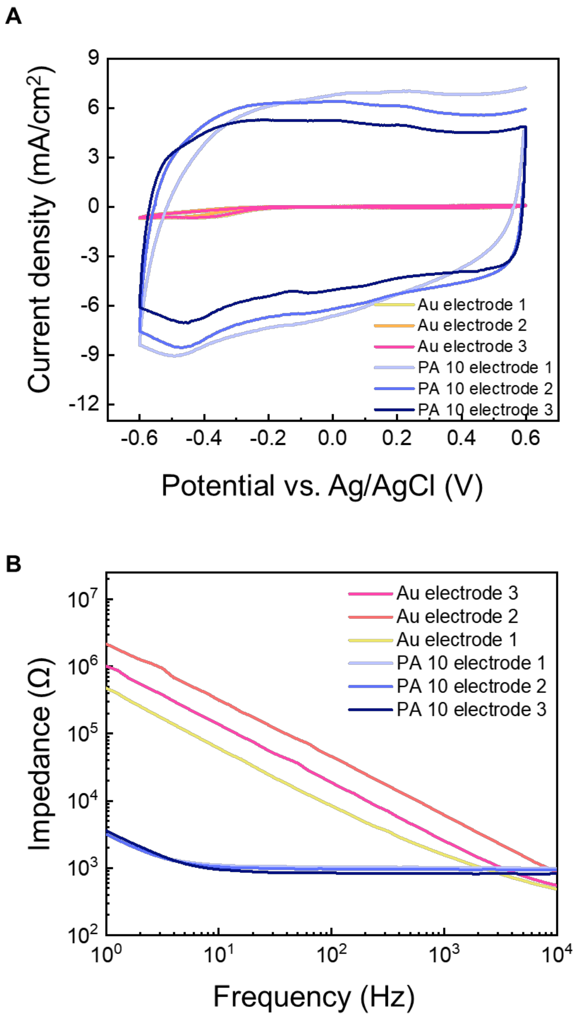


**C**

| Gold | Average | S.D |
|---|---|---|
| CSC ($mC/cm^2$) | 0.4137 | 0.0381 |
| Z (kΩ) | 3.417 | 1.139 |

| PA 10 | Average | S.D |
|---|---|---|
| CSC ($mC/cm^2$) | 32.13 | 1.514 |
| Z (kΩ) | 0.923 | 0.042 |

**PA 10 was spin coated 3 times for thick electrode (1000 RPM each)**

**Fig. S20. Electrochemical property comparison of PA 10 and Au electrode.**

(**A**) CSC results of PA 10 and Au electrodes with 0.25 $mm^2$ of exposed area. (**B**) Impedance results of PA 10 and Au electrode with 0.25 $mm^2$ of exposed area. (**C**) Average CSC and impedance results.

| Fabrication methods | Additives | Conductivity in aqueous environment | Spatial resolution | Reference |
|---|---|---|---|---|
| Chemical treatment | Poly(ethylene glycol)diglycidyl ether (PEGDE) | 706 S $cm^{-1}$ | N/A | 41 |
| Chemical treatment | Sulfuric acid ($H_2SO_4$) | 8.8 S $cm^{-1}$ | N/A | 9 |
| Molding | Ionic liquid (I·L) | 23 S $cm^{-1}$ | 10 μm | 10 |
| Screen printing | Dimethyl sulfoxide (DMSO) | 40 S $cm^{-1}$ | 400 μm | 13 |
| Photolithography | Ionic liquid (I·L) | 47 S $cm^{-1}$ | 5 μm | 4 |
| Inkjet printing | Ionic liquid (I·L) | 900 S $cm^{-1}$ | 300 μm | 19 |
| Electro-gelation | N/A | 0.23 S $cm^{-1}$ | 100 μm | 11 |
| 3D printing | Dimethyl sulfoxide (DMSO) | 28 S $cm^{-1}$ | 30 μm | 14 |
| **This work (LIPSP)** | **AuNP** | **670 S $cm^{-1}$** | **6 μm** | **N/A** |

**Table S1. Comparison of the LIPSP with preceding studies in terms of the conductivity and spatial resolution in aqueous environments.**

| Fabrication methods | Additives | Detoxification Process | Reference |
|---|---|---|---|
| Chemical treatment | PEGDE | Not required | 41 |
| Chemical treatment | $H_2SO_4$ | Repeated washing with water | 9 |
| Molding | I·L | Hours of washing with water | 10 |
| Screen printing | DMSO | 24 h drying at 60 °C | 13 |
| Photolithography | I·L | Soaking in water for 24 h, water replacement every 8 h | 4 |
| Inkjet printing | I·L | Repeated washing with water | 19 |
| Electrogelation | N/A | Storage in PBS for 1 week for removing metal ions | 11 |
| 3D printing | DMSO | 24 h drying at 60 °C | 14 |
| **This work (LIPSP)** | **AuNP** | **Not required** | **N/A** |

**Table S2. Comparison of the LIPSP with preceding studies in terms of post-processing time.**

**Legend for Supplementary Movie**

**Movie S1. Laser-induced phase separation of PEDOT:PSS**
**Movie S2. Cleaning process**
**Movie S3. Implantation behavior test**
**Movie S4. Neural stimulation**

## REFERENCES AND NOTES


1. H. Yuk, B. Lu, X. Zhao, Hydrogel bioelectronics. *Chem. Soc. Rev.* **48**, 1642–1667 (2019).

2. X. Fan, W. Nie, H. Tsai, N. Wang, H. Huang, Y. Cheng, R. Wen, L. Ma, F. Yan, Y. Xia, PEDOT:PSS for flexible and stretchable electronics: Modifications, strategies, and applications. *Adv. Sci.* **6**, 1900813 (2019).

3. N. Kim, S. Lienemann, I. Petsagkourakis, D. A. Mengistie, S. Kee, T. Ederth, V. Gueskine, P. Leclere, R. Lazzaroni, X. Crispin, K. Tybrandt, Elastic conducting polymer composites in thermoelectric modules. *Nat. Commun.* **11**, 1424 (2020).

4. Y. Liu, J. Liu, S. Chen, T. Lei, Y. Kim, S. Niu, H. Wang, X. Wang, A. M. Foudeh, J. B. Tok, Z. Bao, Soft and elastic hydrogel-based microelectronics for localized low-voltage neuromodulation. *Nat. Biomed. Eng.* **3**, 58–68 (2019).

5. Y. Liu, J. Li, S. Song, J. Kang, Y. Tsao, S. Chen, V. Mottini, K. McConnell, W. Xu, Y. Zheng, J. B.-H. Tok, P. M. George, Z. Bao, Morphing electronics enable neuromodulation in growing tissue. *Nat. Biotechnol.* **38**, 1031–1036 (2020).

6. Z. Aqrawe, J. Montgomery, J. T. Sejdic, D. Svirskis, Conducting polymers for neuronal microelectrode array recording and stimulation. *Sens. Actuators B Chem.* **257**, 753–765 (2018).

7. M. Ganji, E. Kaestner, J. Hermiz, N. Rogers, A. Tanaka, D. Cleary, S. H. Lee, J. Snider, M. Halgren, G. R. Cosgrove, B. S. Carter, D. Barba, I. Uguz, G. G. Malliaras, S. S. Cash, V. Gilja, E. Halgren, S. A. Dayeh, Development and translation of PEDOT:PSS microelectrodes for intraoperative monitoring. *Adv. Funct. Mater.* **28**, 1700232 (2018).

8. U. Lang, N. Naujoks, J. Dual, Mechanical characterization of PEDOT:PSS thin films. *Synth. Met.* **159**, 473–479 (2009).

9. B. Yao, H. Wang, Q. Zhou, M. Wu, M. Zhang, C. Li, G. Shi, Ultrahigh-conductivity polymer hydrogels with arbitrary structures. *Adv. Mater.* **29**, 1700974 (2017).

10. V. R. Feig, H. Tran, M. Lee, Z. Bao, Mechanically tunable conductive interpenetrating network hydrogels that mimic the elastic moduli of biological tissue. *Nat. Commun.* **9**, 2040 (2018).

11. V. R. Feig, H. Tran, M. Lee, K. Liu, Z. Huang, L. Beker, D. G. Mackanic, Z. Bao, An electrochemical gelation method for patterning conductive PEDOT:PSS hydrogels. *Adv. Mater.* **31**, 1902869 (2019).

12. S. Zhang, Y. Chen, H. Liu, Z. Wang, H. Ling, C. Wang, J. Ni, B. C. Saltik, X. Wang, X. Meng, H. J. Kim, A. Baidya, S. Ahadian, N. Ashammakhi, M. R. Dokmeci, J. T. Sejdic, A. Khademhosseini, Room-temperature-formed PEDOT:PSS hydrogels enable injectable, soft, and healable organic bioelectronics. *Adv. Mater.* **32**, 1904752 (2020).

13. B. Lu, H. Yuk, S. Lin, N. Jian, K. Qu, J. Xu, X. Zhao, Pure PEDOT:PSS hydrogels. *Nat. Commun.* **10**, 1043 (2019).

14. H. Yuk, B. Lu, S. Lin, K. Qu, J. Xu, J. Luo, X. Zhao, 3D printing of conducting polymers. *Nat. Commun.* **11**, 1604 (2020).

15. M. S. Mahajan, D. M. Marathe, S. S. Ghosh, V. Ganesan, J. V. Sali, Changes in in-plane electrical conductivity of PEDOT:PSS thin films due to electric field induced dipolar reorientation. *RSC Adv.* **5**, 86393–86401 (2015).

16. K. Lim, J. Kang, S. Jung, S. Lee, J. Park, D. G. Kim, Y. C. Kang, Improving electrical conductivity of PEDOT:PSS with phase separation by applying electric fields. *Bull. Korean Chem. Soc.* **39**, 469–476 (2018).

17. N. Chaturvedi, F. Alam, S. K. Swami, V. Dutta, Effect of electric field on the spray deposited poly (3,4-ethylenedioxythiophene): Poly(styrenesulfonate) layer and its use in organic solar cell. *J. Appl. Phys.* **114**, 184501 (2013).

18. L. D. Garma, L. M. Ferrari, P. Scognamiglio, F. Greco, F. Santoro, Inkjet-printed PEDOT:PSS multi-electrode arrays for low-cost in vitro electrophysiology. *Lab Chip* **19**, 3776–3786 (2019).

19. M. Y. Teo, N. RaviChandran, N. Kim, S. Kee, L. Stuart, K. C. Aw, J. Stringer, Direct patterning of highly conductive PEDOT:PSS/ionic liquid hydrogel via microreactive inkjet printing. *ACS Appl. Mater. Interfaces* **11**, 37069–37076 (2019).

20. D. N. Heo, S. J. Lee, R. Timsina, X. Qiu, N. J. Castro, L. G. Zhang, Development of 3D printable conductive hydrogel with crystallized PEDOT: PSS for neural tissue engineering. *Mater. Sci. Eng. C* **99**, 582–590 (2019).

21. C. Yun, J. W. Han, S. Kim, D. C. Lim, H. Jung, S. H. Lee, J. W. Jang, S. Yoo, K. Leo, Y. H. Kim, Generating semi-metallic conductivity in polymers by laser-driven nanostructural reorganization. *Mater. Horiz.* **6**, 2143–2151 (2019).

22. S. Chae, K. H. Jo, S. W. Lee, H. S. Keum, H. J. Kim, J. Choi, H. H. Lee, Selective chain alignment of conducting polymer blend films by an ultrafast laser. *Macromol. Chem. Phys.* **217**, 537–542 (2016).

23. C. Qin, X. Zhang, W. He, G. Zhang, R. Chen, Y. Gao, L. Xiao, S. Jia, Continuous-wave laser-induced welding and giant photoluminescence enhancement of Au nanospheres. *Opt. Express* **27**, 2886–2898 (2019).

24. I. Song, N. Y. Park, G. S. Jeong, J. H. Kang, J. H. Seo, J.-Y. Choi, Conductive channel formation for enhanced electrical conductivity of PEDOT:PSS with high work-function. *Appl. Surf. Sci.* **529**, 147176 (2020).

25. S. M. Kim, C. H. Kim, Y. Kim, N. Kim, W. J. Lee, E. H. Lee, D. Kim, S. Park, K. Lee, J. Rivnay, M. H. Yoon, Influence of PEDOT:PSS crystallinity and composition on electrochemical transistor performance and long-term stability. *Nat. Commun.* **9**, 3858 (2018).

26. A. Hu, L. Tan, X. Hu, L. Hu, Q. Ai, X. Meng, L. Chen, Y. Chen, Crystallization and conformation engineering of solution-processed polymer transparent electrodes with high conductivity. *J. Mater. Chem. C.* **5**, 382–389 (2017).

27. J. Nevrela, M. Micjan, M. Novota, S. Kovacova, M. Pavuk, P. Juhasz, J. Kovac, J. Jakabovic, M. Weis, Secondary doping in poly(3,4-ethylenedioxythiophene):Poly(4-styrenesulfonate) thin films. *J Polym Sci B* **53**, 1139–1146 (2015).

28. Q. Li, J. Yang, S. Chen, J. Zou, W. Xie, X. Zeng, Highly conductive PEDOT: PSS transparent hole transporting layer with solvent treatment for high performance silicon/organic hybrid solar cells. *Nanoscale Res. Lett.* **12**, 506 (2017).

29. L. Qian, H. Zhao, Nanoindentation of soft biological materials. *Micromachines.* **9**, 654 (2018).

30. J. H. Kim, A. Nizami, Y. Hwangbo, B. Jang, H. J. Lee, C. S. Woo, S. Hyun, T. S. Kim, Tensile testing of ultra-thin films on water surface. *Nat. Commun.* **4**, 2520 (2013).

31. R. Akhtar, M. J. Sherratt, J. K. Cruickshank, B. Derby, Characterizing the elastic properties of tissues. *Mater. Today* **14**, 96–105 (2011).

32. K. Feron, R. Lim, C. Sherwood, A. Keynes, A. Brichta, P. C. Dastoor, Organic bioelectronics: Materials and biocompatibility. *Int. J. Mol. Sci.* **19**, 2382 (2018).

33. L. D. Medinaceli, W. J. Freed, R. J. Wyatt, An index of the functional condition of rat sciatic nerve based on measurements made from walking tracks. *Exp. Neurol.* **77**, 634–643 (1982).

34. M. J. Piesla, L. Leventhal, B. W. Strassle, J. E. Harrison, T. A. Cummons, P. Lu, G. T. Whiteside, Abnormal gait, due to inflammation but not nerve injury, reflects enhanced nociception in preclinical pain models. *Brain Res.* **1295**, 89–98 (2009).

35. A. G. Sulser, J. Wang, B. N. Queenan, M. Avoli, S. Vicini, R. Dzakpasu, Hippocampal neuron firing and local field potentials in the in vitro 4-aminopyridine epilepsy model. *J. Neurophysiol.* **108**, 2568–2580 (2012).

36. V. Viswam, M. E. J. Obien, F. Franke, U. Frey, A. Hierlemann, Optimal electrode size for multi-scale extracellular-potential recording from neuronal assemblies. *Front. Neurosci.* **13**, 385 (2019).

37. A. O. Rosario, H. Adeli, Brain-computer interface technologies: From signal to action. *Rev. Neurosci.* **24**, 537–552 (2013).

38. A. H. Bozer, M. L. Uhelski, A. L. Li, Extrapolating meaning from local field potential recordings. *J. Integr. Neurosci.* **16**, 107–126 (2017).

39. I. Khalilov, V. Dzhala, Y. B. Ari, R. Khazipov, Dual role of GABA in the neonatal rat hippocampus. *Dev. Neurosci.* **21**, 310–319 (1999).

40. R. Stoop, F. Conquet, B. Zuber, L. L. Voronin, E. Pralong, Activation of metabotropic glutamate 5 and NMDA receptors underlies the induction of persistent bursting and associated long-lasting changes in CA3 recurrent connections. *J. Neurosci.* **23**, 5634–5644 (2003).

41. M. Solazzo, K. Krukiewicz, A. Zhussupbekova, K. Fleischer, M. J. Biggs, M. G. Monaghan, PEDOT: PSS interfaces stabilised using a PEGylated crosslinker yield improved conductivity and biocompatibility. *J. Mater. Chem. B* **7**, 4811–4820 (2019).
